# Glutamatergic neurons of the gigantocellular reticular nucleus shape locomotor pattern and rhythm in the freely behaving mouse

**DOI:** 10.1371/journal.pbio.2003880

**Published:** 2019-04-24

**Authors:** Maxime Lemieux, Frederic Bretzner

**Affiliations:** 1 Centre de Recherche du CHU de Québec, CHUL-Neurosciences, Québec (QC), Canada; 2 Faculty of Medicine, Department of Psychiatry and Neurosciences, Université Laval, Québec (QC), Canada; Emory University School of Medicine, United States of America

## Abstract

Because of their intermediate position between supraspinal locomotor centers and spinal circuits, gigantocellular reticular nucleus (GRN) neurons play a key role in motor command. However, the functional contribution of glutamatergic GRN neurons in initiating, maintaining, and stopping locomotion is still unclear. Combining electromyographic recordings with optogenetic manipulations in freely behaving mice, we investigate the functional contribution of glutamatergic brainstem neurons of the GRN to motor and locomotor activity. Short-pulse photostimulation of one side of the glutamatergic GRN did not elicit locomotion but evoked distinct motor responses in flexor and extensor muscles at rest and during locomotion. Glutamatergic GRN outputs to the spinal cord appear to be gated according to the spinal locomotor network state. Increasing the duration of photostimulation increased motor and postural tone at rest and reset locomotor rhythm during ongoing locomotion. In contrast, photoinhibition impaired locomotor pattern and rhythm. We conclude that unilateral activation of glutamatergic GRN neurons triggered motor activity and modified ongoing locomotor pattern and rhythm.

## Introduction

Locomotion results from an interplay between the spinal circuit that generates locomotor pattern and rhythm, the sensory inputs from the peripheral afferents that shape motor responses, and the supraspinal descending inputs from the brainstem that send the locomotor command. As demonstrated by reversible cooling [1–3] or pharmacological blockade [4, 5], the descending locomotor command depends on the integrity of the brainstem and its ipsilateral reticulospinal pathway. The firing pattern of reticular and reticulospinal neurons of the gigantocellular reticular nucleus (GRN) is in phase with locomotor activity [6–8]; firing increases while walking uphill (pitch tilt), during left or right roll tilt [9], or while stepping over obstacles during locomotion [10]. Moreover, electrical microstimulations within the GRN also modulate locomotor pattern according to the phase of the step cycle [11–14] in addition to resetting locomotor rhythm [12, 14]. While it is clear that descending GRN neurons are important to motor command in initiating, maintaining, and terminating locomotion, the exact contribution of glutamatergic neurons remains uncertain.

Recent advances in mouse genetics have revealed the importance of a discrete population of brainstem neurons to locomotion. Glutamatergic GRN neurons genetically identified as V2a have been described as firing tonically, projecting to the spinal cord, and relaying functional inputs from supraspinal locomotor centers in addition to increasing a marker of cell activity, c-FOS, following an episode of locomotion [15]. Recently, optogenetic activation of V2a GRN neurons has been shown to prolong the stance phase in freely walking mice and to abolish flexor-related locomotor-like activity in isolated neonatal preparations [16], thus leading to a proposal to name V2a GRN neurons as “stop cells.” Although most V2a neurons are glutamatergic [15–17], they are not all reticulospinal [15, 18], and presumably not all glutamatergic GRN neurons are V2a. Given the large proportion of GRN neurons expressing vesicular glutamate transporter 2 (VGluT2) mRNA and their locomotor-related effects, it is surprising that glutamatergic GRN neurons only induce head turnings in open field [19], thus raising questions about the functional contribution of glutamatergic GRN neurons to motor command.

Combining electromyographic (EMG) recordings with unilateral optogenetic manipulations, we evaluated the necessity and sufficiency of glutamatergic GRN neurons to initiate, maintain, and/or stop motor and locomotor activity in the freely behaving mouse. Short-pulse photostimulation (10 ms) increased motor tone in the resting mouse and modified locomotor activity according to the phase of the step cycle in the walking mouse, thus contributing to shaping locomotor pattern. Although long photostimulation failed to initiate locomotion, it reset locomotor rhythm, suggesting that it accesses the rhythm generator. In contrast, long photoinhibition altered locomotor pattern and rhythm. Therefore, our findings reveal that glutamatergic GRN neurons, as a descending excitatory pathway, play a key role in motor control and locomotion.

## Results

**Fig 1:**
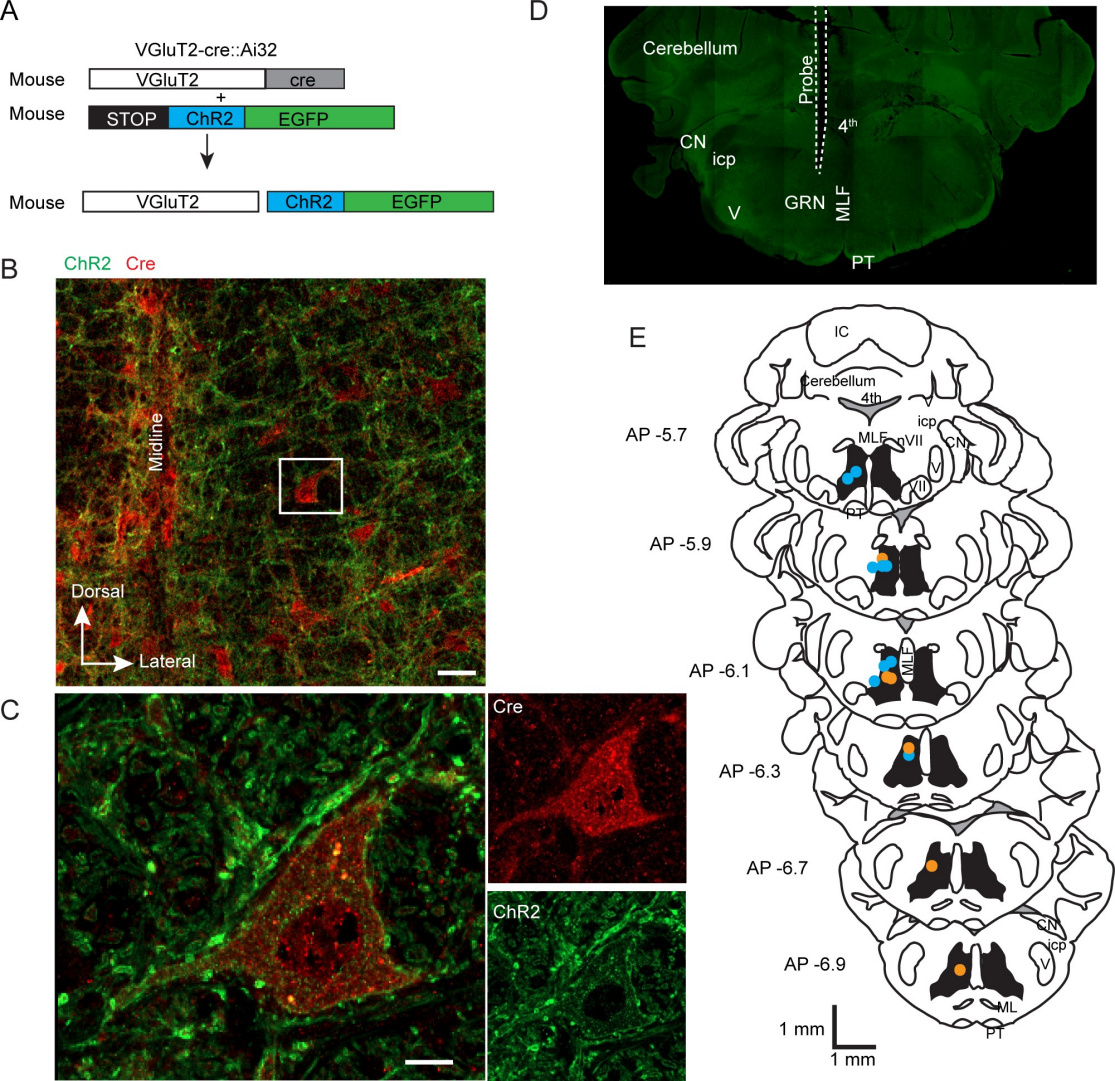
Experimental design. (A) Experimental design using genetic expression of ChR2. (B) Low-magnification image of the GRN. Cre-positive neurons are in red (VGluT2), and the ChR2–EYFP fusion protein signal is in green. The neuron in the white box is shown at higher magnification in panel C. Scale bar is 50 μm. (C) Cre-positive neuron (red) in the GRN. ChR2–EYFP is observed on the membrane of the Cre-positive neuron but also in the surrounding space (presumably dendrites and axons of other neurons). Scale bar is 10 μm. (D) Brainstem section from a VGluT2-cre::Ai32 mouse showing the track of the optical probe. (E) Schema of brain sections illustrating the location of single fibers (blue circle) and array of triple fibers (orange circle). AP, anteroposterior; ChR2, channelrhodopsin-2; CN, cochlear nucleus EGFP, enhanced green fluorescent protein; EYFP, enhanced yellow fluorescent protein; GRN, gigantocellular reticular nucleus; IC, inferior colliculus; icp, inferior cerebellar peduncle; ML, medial lemniscus; MLF, medial longitudinal fascicle; PT, pyramidal tract; V, trigeminal nucleus; VGluT2, vesicular glutamate transporter 2.

Using VGluT2-cre mice, channelrhodopsin-2 (ChR2) was conditionally expressed in glutamatergic GRN neurons by backcrossing transgenic mice (Fig 1A, *n* = 16 VGluT2-cre and Ai32-ChR2 mice). Two types of optical probes were inserted above the GRN: single fibers of 100 μm in diameter or arrays of 3 fibers each spaced by 200 μm (S1 Fig, *n* = 6 mice for single fibers and *n* = 10 for arrays). The location of the probe was verified at the end of the experiment (Fig 1D and 1E). The colocalization of VGluT2 mRNA and CRE-expressing neurons has been shown previously [20]. Cre-recombinase signal was distributed throughout the medullary reticular formation and was absent from the pyramidal tract. The ChR2–enhanced yellow fluorescent protein (EYFP) signal (amplified with anti-green fluorescent protein [GFP]) was observed on the membrane of Cre-positive neurons but also in what we presume to be dendrites and axons of other Cre-positive neurons (Figs 1B and 1C and S2). VGluT2 GRN neurons were photostimulated upon blue laser illumination (473 nm) delivered through optical probes. According to our calculation, the laser irradiance at the tip of each fiber of the array corresponded to 75% of a single fiber (or an attenuation of 25%).

### Recruitment of GRN neurons and motor responses upon photostimulation

**Fig 2:**
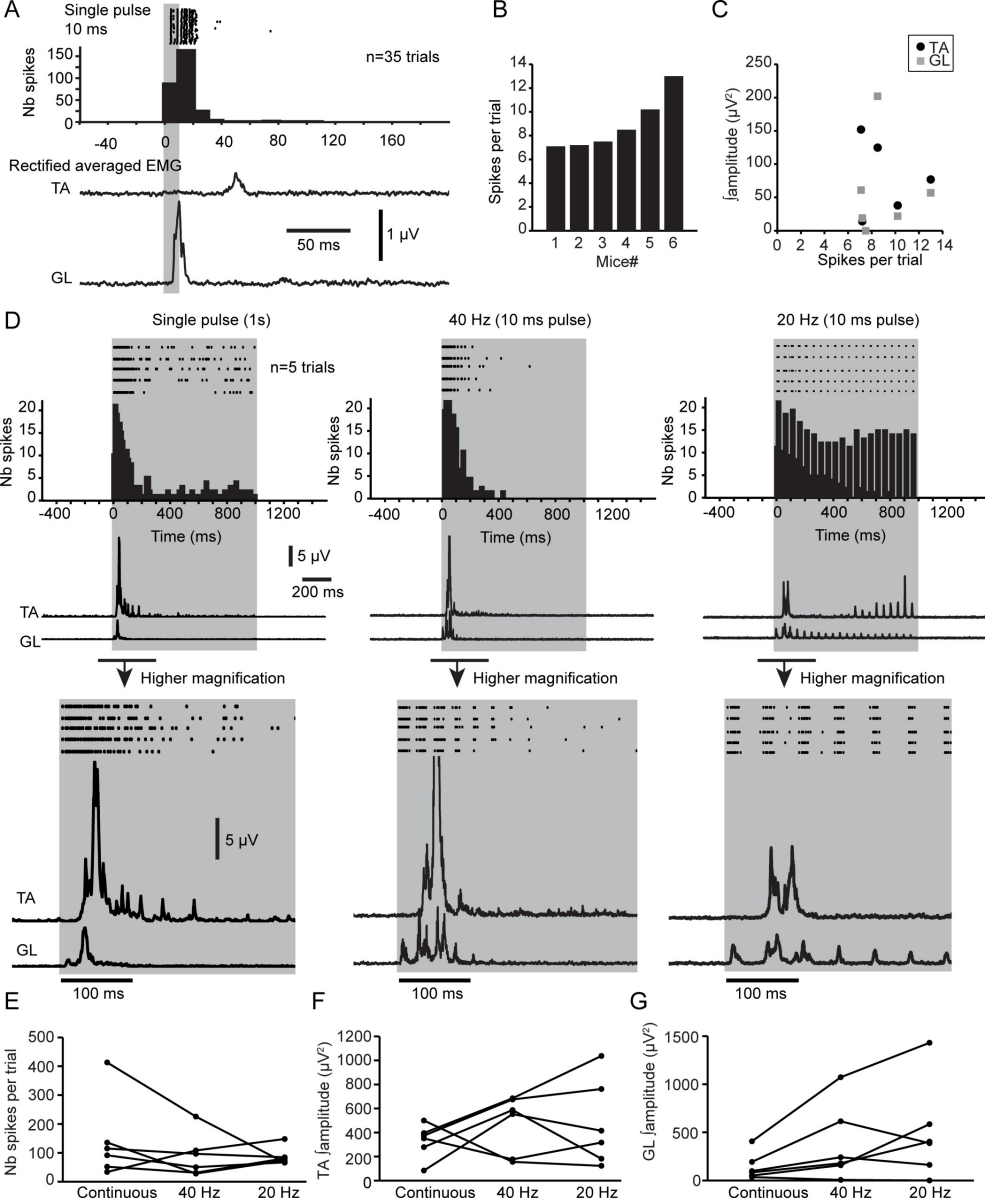
Firing pattern and motor responses evoked upon short-pulse PS of VGluT2 GRN neurons during ketamine–xylazine anesthesia. (A) Raster plot and frequency histogram of the firing of VGluT2 neurons upon short-pulse (10-ms) PS. Rectified and averaged EMG of the TA and the GL are shown below the histogram. (B) Bar graph of the average number of spikes per trial upon short-pulse PS for each mouse. (C) Plot of the integrated amplitude (area) of the TA and the GL versus the average number of spikes per trial. (D) Raster plot and frequency histogram of firing of VGluT2 neurons upon 1-s single pulse (left column) or 1-s train of 10-ms pulses (middle, 40 Hz; right, 20 Hz) along rectified averaged EMG of the TA and the GL. Higher magnifications at the beginning of the PS are at the bottom. (E) Plot of the average number of spikes per trial during 1-s PS (continuous and trains of 40 and 20 Hz). No significant differences between the types of PS: Kruskal-Wallis test, *p* = 0.65. (F) Plot of the integrated amplitude (area) of the TA during 1-s PS (continuous and trains of 40 and 20 Hz). No significant differences between the types of PS: Kruskal-Wallis test, *p* = 0.57. (G) Plot of the integrated amplitude (area) of the GL during 1-s PS (continuous and trains of 40 and 20 Hz). No significant differences between the types of PS: Kruskal-Wallis test, *p* = 0.13. Data can be found in S1 Data. EMG, electromyography; GL, gastrocnemius lateralis; PS, photostimulation; TA, tibialis anterior; VGluT2, vesicular glutamate transporter 2.

We evaluated the firing of VGluT2 GRN neurons under ketamine–xylazine anesthesia. Upon short pulses of 10 ms, 7–13 spikes per trial were recorded in the vicinity of the optical probe (Fig 2A and 2B). The density of spikes was maximal within 10–20 ms and rapidly decreased within 40 ms. In addition, motor responses were evoked in both the ankle dorsiflexor tibialis anterior (TA) and the ankle extensor gastrocnemius lateralis (GL) in the hindlimb ipsilateral to the photostimulation site. The amplitude of motor responses was not correlated with the number of spikes (Fig 2C).

We next evaluated the firing pattern during long photostimulation, either long pulses or trains of 40 or 20 Hz for 1 s (Fig 2D). Long pulses and long trains of 40 Hz evoked the strongest firing pattern and motor responses for the first 200 ms. The firing pattern reliably followed long trains at 20 Hz, but motor responses did not. The number of spikes and motor responses were not affected according to the types of photostimulation (continuous versus trains of 20 or 40 Hz, Fig 2E and 2G). Using 6-cyano-7-nitroquinoxaline-2,3-dione (CNQX) and (2R)-amino-5-phosphonovaleric acid (AP-5) to block the local glutamatergic transmission, we found no significant changes in the firing pattern or motor responses (S3 Fig), thus suggesting that motor effects were induced primarily by direct activation of VGluT2 GRN neurons in the vicinity of the optical probe and not by spatiotemporal recruitment of the local network.

### Short-pulse photostimulation evokes motor responses at rest

**Fig 3:**
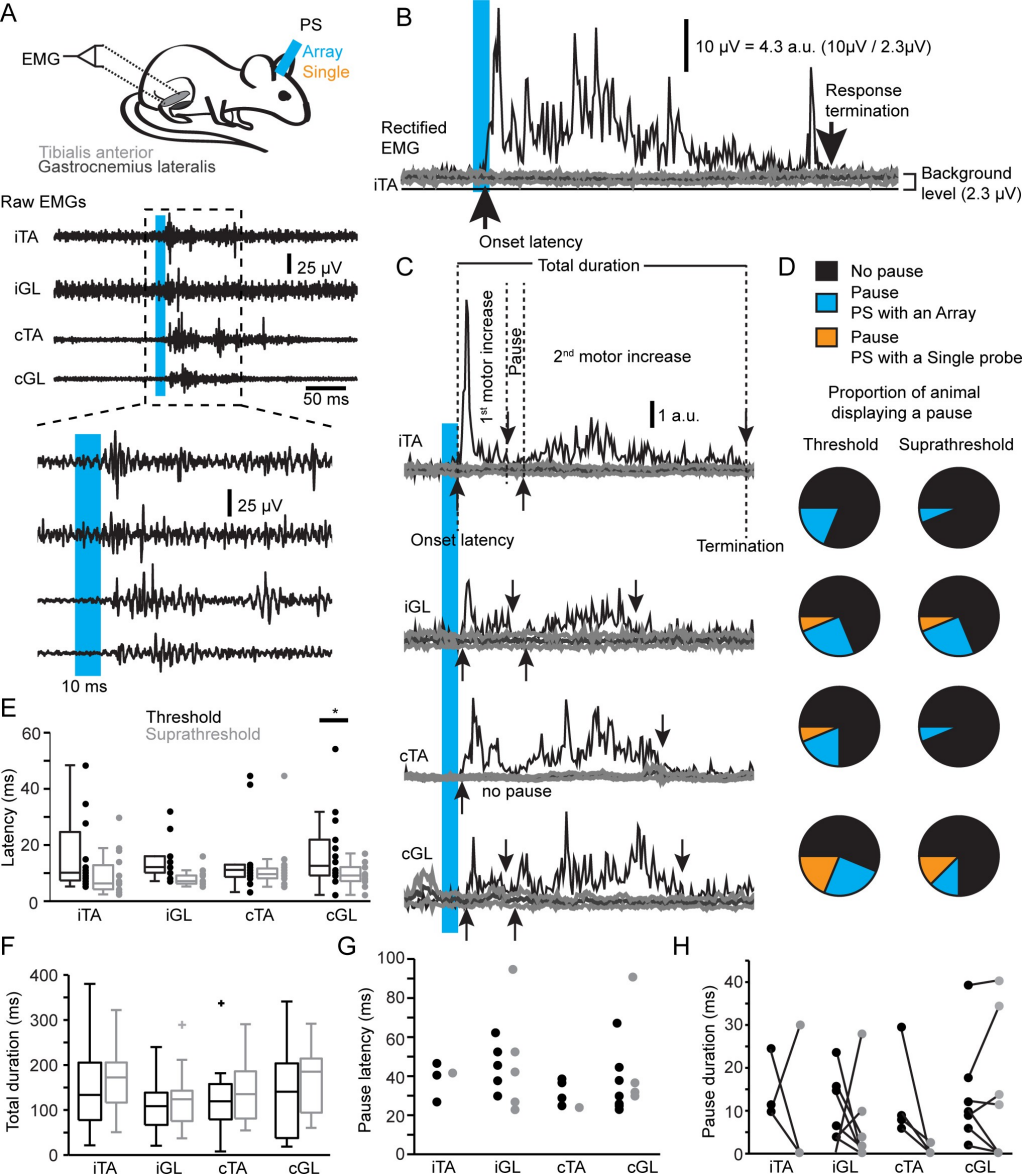
Motor responses evoked by short-pulse PS of VGluT2 GRN neurons in the resting mouse. (A) Experimental design (top) to photostimulate and record EMGs from the ankle flexor (TA) and extensor (GL). EMG traces of bilateral TAs and GLs at low (middle) and high (bottom) temporal resolution. (B) Rectified EMG of the iTA response (black trace, stimulated period) and the background activity (thick gray traces, unstimulated period). Onset latency of the responses is defined as the time when the response signal passes above the 99.95 confidence interval and the termination of the responses as the time when the signal passes below the same confidence interval. Amplitude scale bars indicate normalization procedure. (C) Rectified EMGs normalized by background activity. In this example, the motor response was briefly interrupted by a short pause corresponding to the period between the first downward and second upward arrows. (D) Pie charts of the occurrence of pauses for all muscles during PS at threshold and suprathreshold intensities. (E) Boxplots of motor response latencies at threshold and suprathreshold laser intensities. Paired *t* test, *p < 0.05. (F) Boxplots of motor response total duration at threshold and suprathreshold laser intensities. (G) Plots of pause latency at threshold and suprathreshold laser intensities. Kruskal-Wallis test, *p* = 0.583. (H) Plots of pause duration at threshold and suprathreshold laser intensities. Bars link values from the same animal. No differences between muscles: Kruskal-Wallis test, *p* = 0.583. No differences for each muscle between threshold and suprathreshold intensity (Wilcoxon signed rank): iTA, *p* = 0.75; iGL, *p* = 0.69; cTA, *p* = 0.12; cGL, *p* = 0.94. Data can be found in S1 Data. a.u., arbitrary units; cGL, contralateral GL; cTA, contralateral TA; EMG, electromyography; GL, gastrocnemius lateralis; GRN, gigantocellular reticular nucleus; iGL, ipsilateral GL; iTA, ipsilateral TA; PS, photostimulation; TA, tibialis anterior; VGluT2, vesicular glutamate transporter 2.

Because we were interested in evaluating the contribution of VGluT2 GRN neurons to muscle tone in the resting mouse, we recorded motor responses of the TA and GL in both hindlimbs upon short-pulse photostimulation (10-ms pulse duration) in the animal at rest (Fig 3A). The threshold for consistently evoking motor responses in at least one muscle (usually the ipsilateral TA [iTA]) was 383.9 ± 215.8 mW/mm^2^ for the single fiber and slightly lower for the array (293.4 ± 148.7 mW/mm^2^, Mann-Whitney, *p* = 0.1019). In all cases, short-pulse photostimulation evoked motor responses in all recorded muscles at suprathreshold (laser intensity 10% above threshold). The onset (response latency) and termination of the response were defined as the times when the response passed respectively above and below a threshold based on the background activity level (Fig 3B). The latency of motor responses did not differ between muscles, and increasing the laser intensity decreased latency only in the contralateral GL (cGL) (Fig 3E). Regarding the responsiveness of muscles, we found that about half of GRN sites elicited motor responses first in the iTA (*n* = 7/15), a third in the contralateral TA (cTA; *n* = 5/15), and seldom in extensors (*n* = 3/15 in the cGL and *n* = 0/15 in the ipsilateral GL [iGL]). Overall, short-latency motor responses were evoked in all muscles in a range (>6 ms) suggesting polysynaptic connectivity to the lumbar motoneuronal pool.

In some mice, motor responses were characterized by an initial increase in EMG activity of short latency and short duration, followed by a short pause (latency: 22.8 to 94.6 ms, Fig 3G; duration: 1.8 to 40.3 ms, Fig 3H) and then followed by a second motor increase (Fig 3C). Pauses were observed more often in GLs than in TAs and were more often elicited by optical arrays. Increasing the laser intensity decreased the occurrence of pauses (Fig 3D). Because pauses were not observed in all animals and were always followed by a second motor increase of longer duration, we considered both excitatory increases as a single motor response.

**Fig 4:**
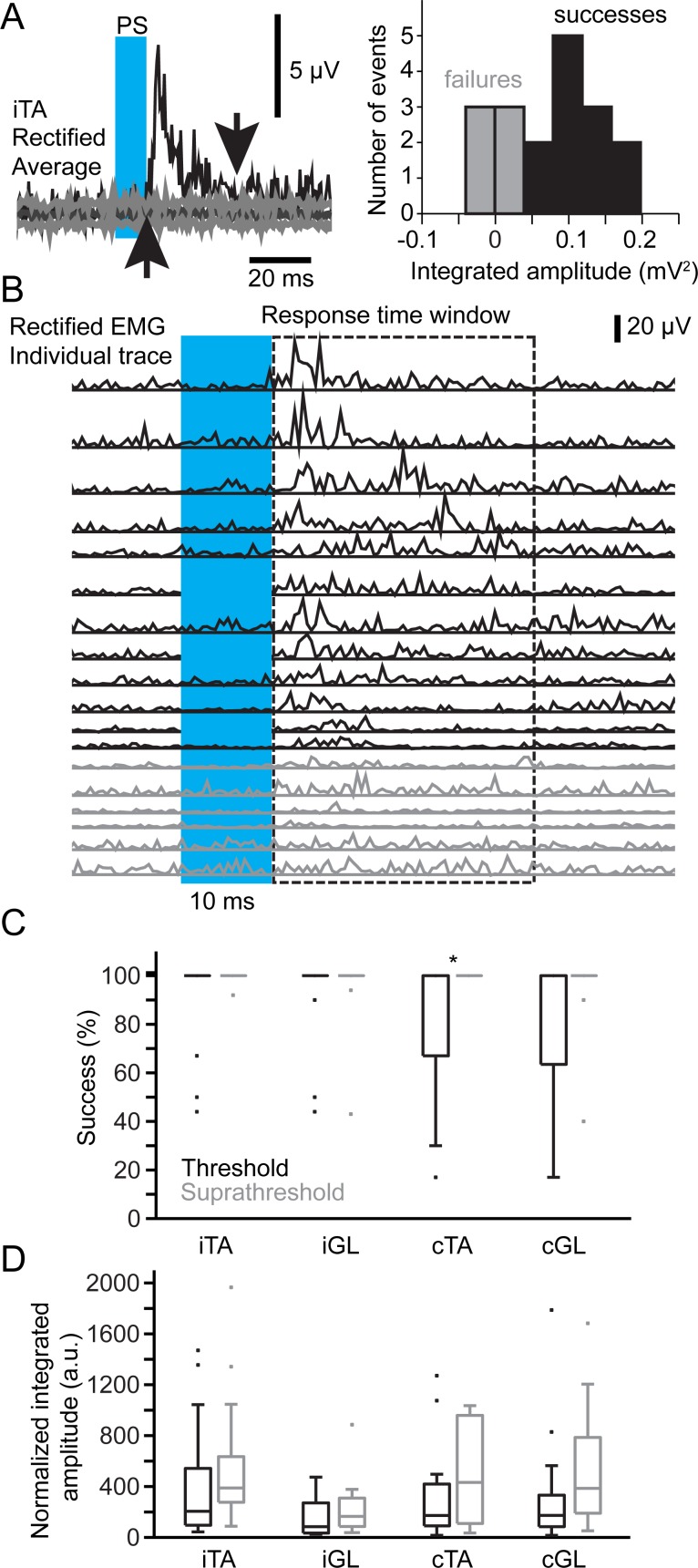
Success and integrated amplitude of motor responses evoked by short-pulse PS of VGluT2 GRN neurons in the resting mouse. (A) Left, examples of rectified averaged EMG response of the iTA to photostimulation. Distribution of EMG responses (middle) from a single animal photostimulated at threshold (4.8 mW). Right, failures are in gray, successes in black. (B) Individual traces of the example shown in (A). The color of traces matches the histogram in (A). The response time window corresponds to the period delimited in (A) by the two arrows. (C) Boxplot of success rate (in %) of motor responses to PS at and above threshold. Wilcoxon signed rank test: *p-value < 0.05 (D) Boxplot of integrated amplitude of motor responses after PS at and above threshold. Data can be found in S1 Data. a.u., arbitrary units; cGL, contralateral gastrocnemius lateralis; cTA, contralateral tibialis anterior; EMG, electromyographic; GRN, gigantocellular reticular nucleus; iGL, ipsilateral gastrocnemius lateralis; iTA, ipsilateral tibialis anterior; PS, photostimulation; VGluT2, vesicular glutamate transporter 2.

There were no differences between muscles in terms of the duration of responses (Fig 3F, Kruskal-Wallis test, *p* = 0.68 at threshold and *p* = 0.25 at suprathreshold intensity). The success rate was not significantly different between muscles (Fig 4C, Kruskal-Wallis test, *p* = 0.55 at threshold and *p* = 0.49 at suprathreshold) and increased with photostimulation intensity only in the cTA (Wilcoxon signed rank test, *p* = 0.022). We also found that all sampled muscles displayed similar levels of integrated amplitude normalized by background activity (Fig 4D, Kruskal-Wallis test, *p* = 0.29 at threshold and *p* = 0.058 at suprathreshold). For each muscle, increasing intensity did not significantly increase the amplitude (Wilcoxon signed rank test: iTA, *p* = 0.45; iGL, *p* = 0.41; cTA, *p* = 0.14; cGL, *p* = 0.20). Therefore, short-pulse photostimulation of VGluT2 GRN neurons recruited bilateral flexor and extensor muscles in the mouse at rest.

### Motor synergy in the mouse at rest

**Fig 5:**
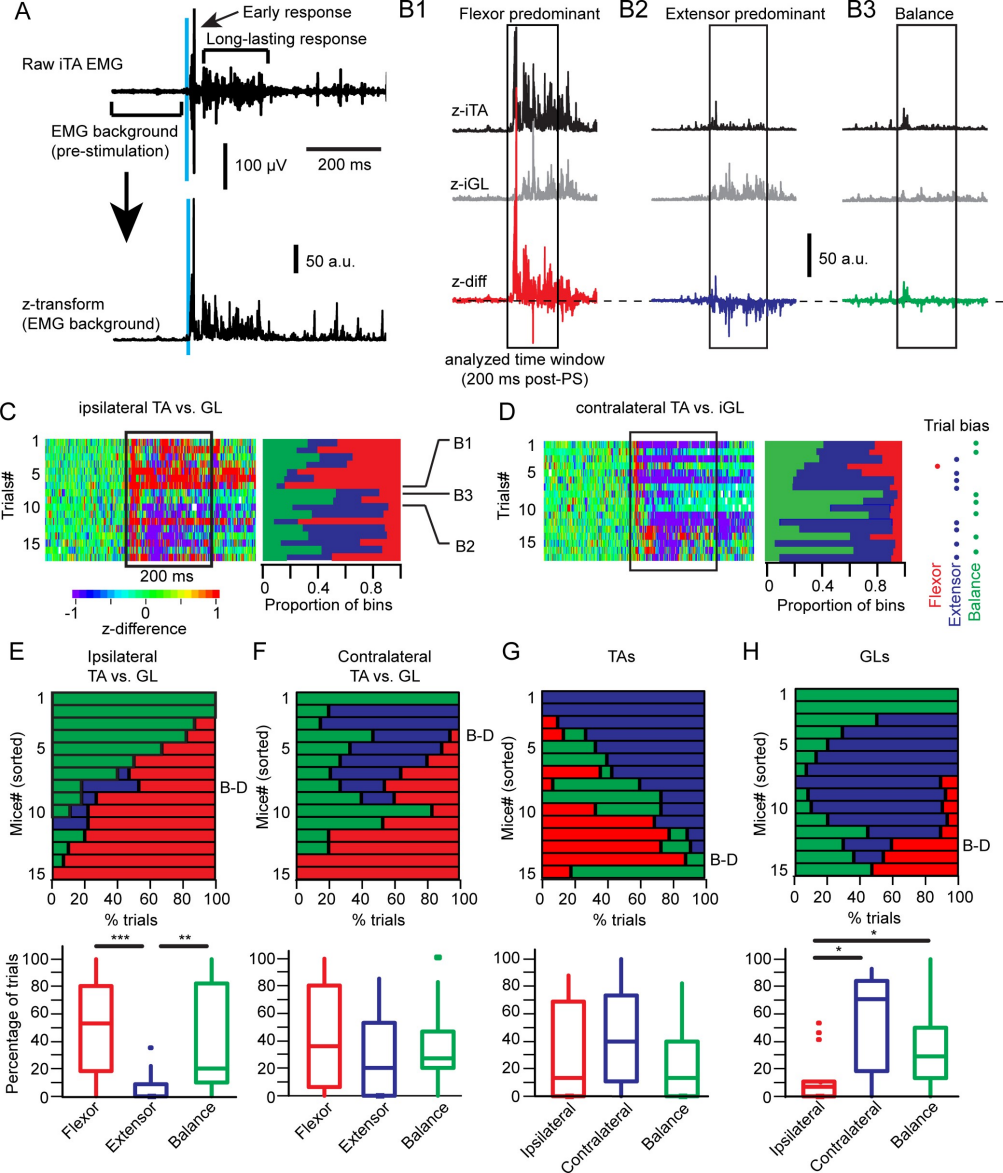
Synergistic motor responses evoked by short-pulse PS in the resting mouse. (A) Top, example of iTA motor response to a 10-ms PS (light blue bar) highlighting early and long-lasting motor responses. Bottom, z-transformation of the raw EMG trace. Average and standard deviation for z-transformation were computed with the EMG background (200 ms prestimulation). (B) Examples of computation to obtain a difference of z-transformed traces of the iTA and iGL (z-diff = ziTA − ziGL). An example is shown for a trial biased toward a flexor activity (B1) or an extensor activity (B2) or a balanced activity between both muscles. (C) Right, color-coded matrix of the z-diff between the iTA and iGL. The abscissa represents time (bin = 1 ms), and each row (ordinates) is a trial. The box starts at PS onset, ends 200 ms later, and delimits the analyzed time window. Left, color-coded matrix of the proportion of bins with a flexor bias (red, z-diff > 1), an extensor bias (blue, z-diff < −1) or a balanced activity (green, −1 > z-diff < 1). (D) Right, color-coded matrix as in (C) for the difference between the cTA and cGL. Middle, color-coded matrix of the proportion of bins with a bias as described in (C). Left, we attributed a bias label based on the dominant condition (flexor bias, extensor bias, or balance) for each trial. (E-H) Top, color-coded matrices of the percentage of trials with a flexor bias, an extensor bias, or a balanced activity (abscissa) for each animal (row in ordinate). The percentage of trials was obtained with the analysis presented in (D). Bottom; boxplots of the percentage of trials are presented at the top to better illustrate the median and statistical differences (Kruskal-Wallis and post hoc Tukey HSD test, *p < 0.05, **p < 0.01, ***p < 0.001). The relationship was investigated between iTA and iGL (E), cTA and cGL (F), left–right TAs (G), and left–right GLs. Data can be found in S1 Data. a.u., arbitrary units; cGL, contralateral GL; cTA, contralateral TA; EMG, electromyographic; GL, gastrocnemius lateralis; GRN, gigantocellular reticular nucleus; HSD, honest significant difference; iGL, ipsilateral GL; iTA, ipsilateral TA; PS, photostimulation; TA, tibialis anterior; z-diff, z-difference.

To assess synergistic motor activity in the animal at rest, we photostimulated glutamatergic GRN neurons at suprathreshold intensity (Fig 5A). Z-difference (z-diff) traces were obtained by subtracting the z-transformed iGL rectified EMG signal from the equally processed iTA signal. Z-transformation was obtained by subtracting the average and dividing by the standard deviation evaluated from the pre-photostimulation period (background). The differences of normalized motor responses were computed on a trial-by-trial basis (Fig 5B, z-diff). When we compared the ipsilateral flexor and extensor (Fig 5B and 5C), some trials were characterized by a flexor-predominant (Fig 5B1), extensor-predominant (Fig 5B2), or balanced (and/or weak) activity (Fig 5B3) in the same animal. To provide a portrait of the relationship between pairs of muscles for all animals (Fig 5E–5H), we identified the bias toward flexor, extensor, or balance for each pair of muscles for each trial (as shown on the right in Fig 5D). When we compared the iTA and iGL, a flexor bias or a balanced activity was observed (Fig 5E), respectively indicating a flexion or a co-contraction. The distribution of biased trials was rather uniform on the contralateral side (Fig 5F). Whereas no predominant bias was observed between the left and right TA (Fig 5G), the cGL was predominant over or balanced with the ipsilateral one (Fig 5H). Overall, VGluT2 GRN neurons evoked a co-contraction outlasting short-pulse photostimulation with an ipsilateral flexor-predominant activity and a contralateral extensor-predominant activity in the animal at rest.

### Short-pulse photostimulation modifies locomotor pattern

**Fig 6:**
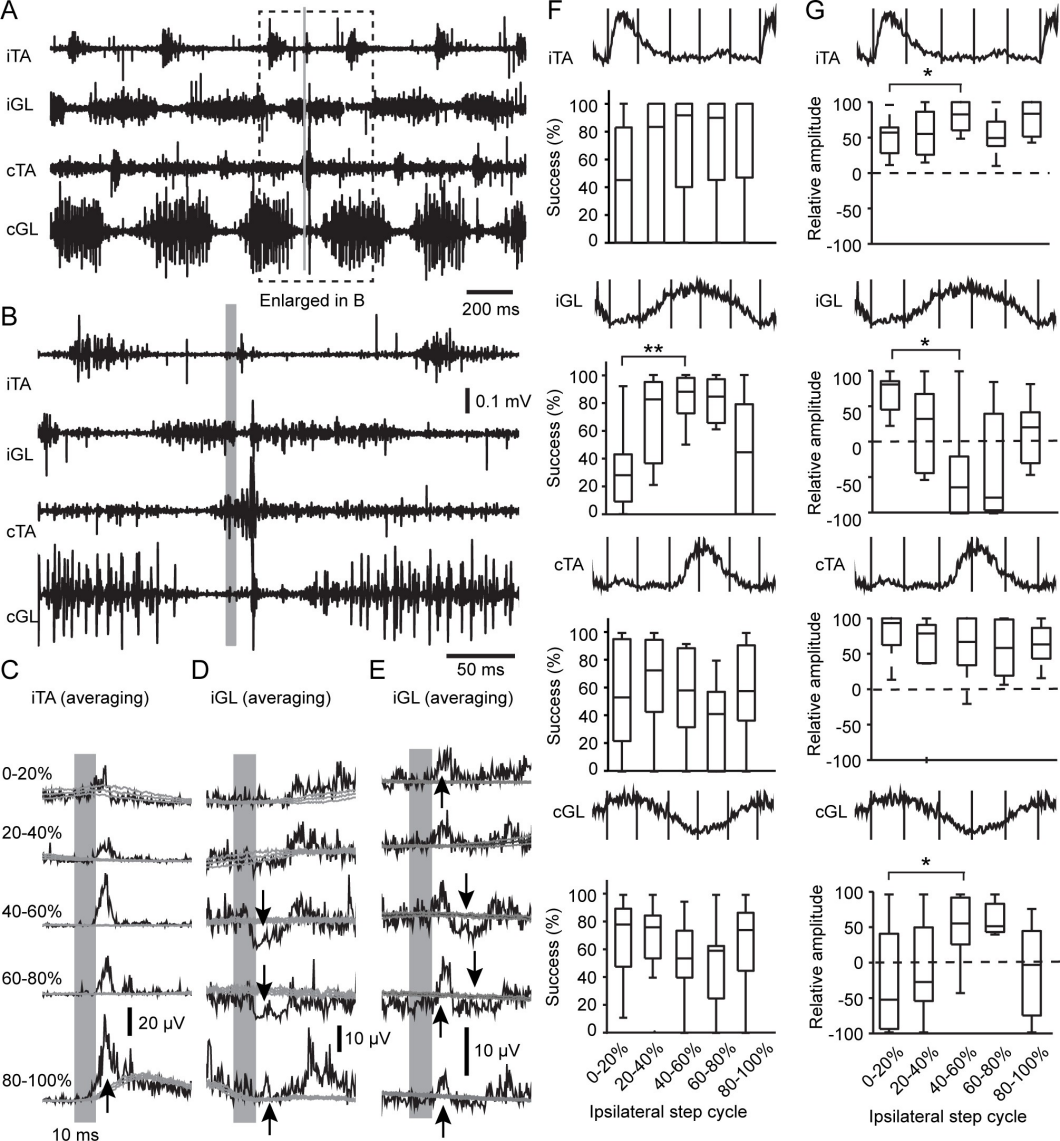
Short-pulse photostimulation of VGluT2 GRN neurons in the freely walking mouse. (A) Raw EMGs of the iTA, iGL, cTA, and cGL during treadmill locomotion at 20 cm/s. Note alternation between flexor and extensor on each side and between homonymous muscles of both sides. (B) Higher temporal resolution of the period underlined in (A). Period highlighted in gray corresponds to the photostimulation. (C) Averaged rectified EMG response of the iTA for each of the 5 periods of the step cycle. Upward arrows indicate a motor increase, and downward arrows indicate a motor decrease. (D, E) Averaged rectified EMG response of the iGL. The example in (D) is from the same animal as in (C). Note inhibition of the iGL during the presumed stance phase (40%–80%). The example in (E) is from another animal to show an increase in phase 0%–20% that is absent in the example in (D) because of a low success rate. (F) Boxplots of the success rate of the EMG responses to photostimulation according to the period of the step cycle. Averaged rectified EMG bursts are displayed above boxplots. ANOVA test: iTA, *p* = 0.28; iGL, *p* = 0.012; cTA, *p* = 0.34; cGL, *p* = 0.31. Post hoc multiple comparisons with *t* test with Bonferroni correction: *p < 0.05, **p < 0.01, ***p < 0.001. (G) Boxplots of the relative amplitude. Same presentation as in (F). ANOVA test: iTA, *p* = 0.032; iGL, *p* = 0.012; cTA, *p* = 0.32; cGL, *p* = 0.003. Post hoc multiple comparisons with *t* test with Bonferroni correction. Data can be found in S1 Data. cGL, contralateral gastrocnemius lateralis; cTA, contralateral tibialis anterior; EMG, electromyography; GRN, gigantocellular reticular nucleus; iGL, ipsilateral gastrocnemius lateralis; iTA, ipsilateral tibialis anterior; VGluT2, vesicular glutamate transporter 2.

Having shown the recruitment of hindlimb muscles by VGluT2 GRN neurons at rest, we next wanted to evaluate the functional contribution of these neurons to locomotor pattern. Short-pulse photostimulation (10 ms) was applied during treadmill locomotion at a suprathreshold laser intensity as evaluated at rest (Fig 6A and 6B, respectively, at low and high temporal resolution). To assess changes in glutamatergic GRN efficacy according to the phase of the step cycle and the level of muscle activity, the step cycle was divided into 5 equal epochs synchronized on the ipsilateral flexor TA, as a proxy of the swing phase. As shown in Fig 6C, short-pulse photostimulation evoked only transient motor increases in the ipsilateral flexor throughout all 5 epochs of the step cycle. In contrast, short-pulse photostimulation evoked a robust decrease in motor responses during the stance phase in the ipsilateral extensor (when the muscle was activated, epoch 40% to 80% of the step cycle, Fig 6D and 6E). A small transient increase was observed when the muscle was relaxed—i.e., at the end of the stance phase (phase 80%–100%) and during the swing phase (epoch 0% to 40% of the step cycle, Fig 6E; success rate was low for the example provided in Fig 6D). The decrease during the activated state was rarely observed in flexors.

The responsiveness (success probability, Fig 6F) and the relative efficacy (relative amplitude of motor responses, Fig 6G) evoked upon short-pulse photostimulation varied slightly according to the phase of the step cycle. However, the relative amplitude of motor responses (Fig 6G) shows that short-pulse photostimulation evoked a statistically significant higher motor increase in the ipsilateral flexor TA while the muscle was relaxed during the stance phase (epoch 40%–60%) than while the muscle was active during the swing phase (epoch 0%–20%). In contrast, short-pulse photostimulation evoked a maximal motor increase in the ipsilateral extensor GL while the muscle was relaxed during the swing and late-stance phases (epochs 0%–20% and 80%–100%, respectively) and a maximal decrease while the muscle was active during the mid-stance phase (epoch 40%–60%). A similar phase-dependent locomotor pattern was found in the contralateral extensor muscle but not in the contralateral flexor (Fig 6G). In summary, VGluT2 GRN neurons drive phase-dependent motor responses with transient excitations in flexor muscles and a mixture of excitation and inhibition in extensor muscles throughout the step cycle.

### Gating of descending glutamatergic GRN inputs during motor and locomotor activity

**Fig 7:**
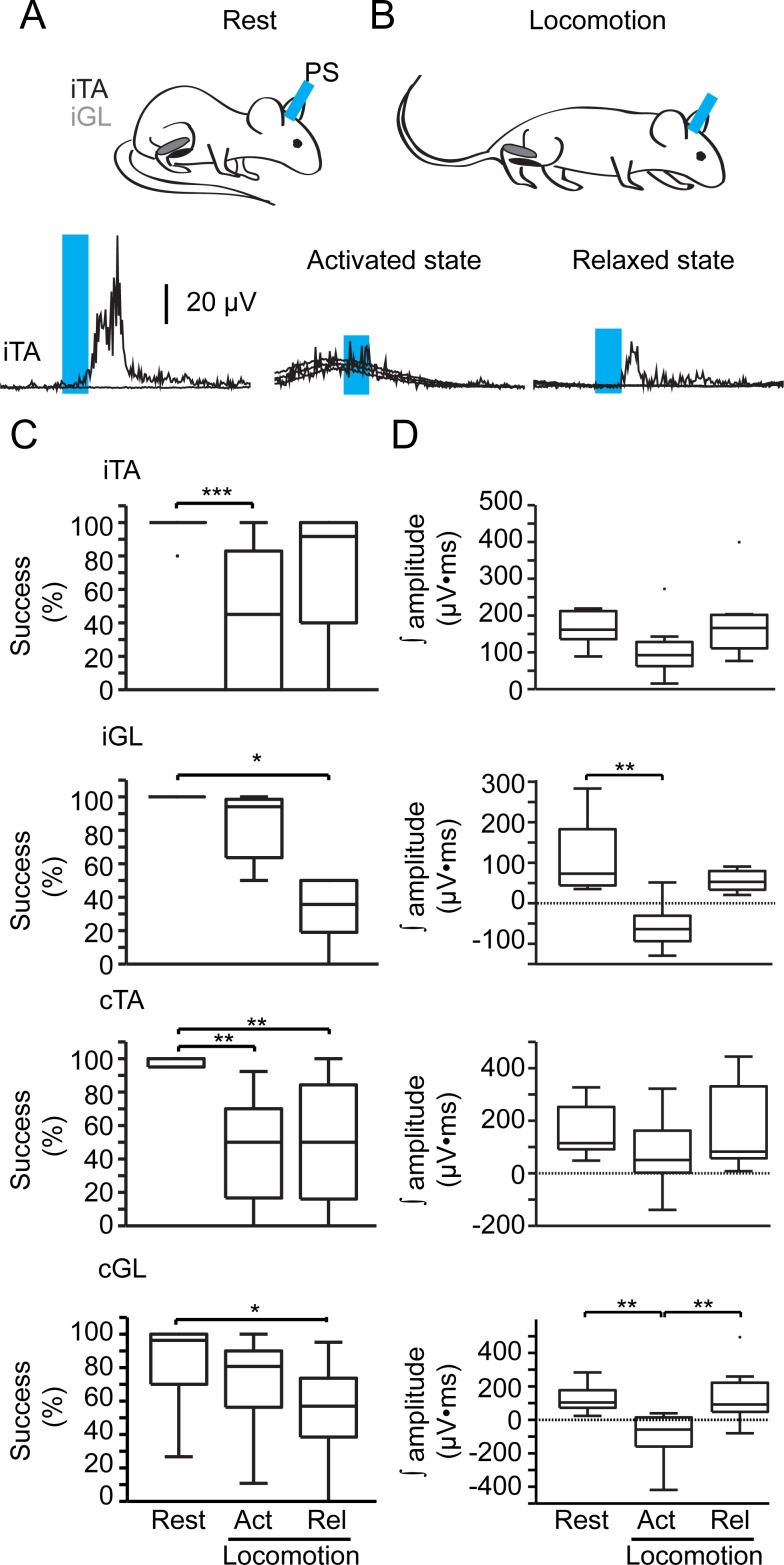
Changes in the success rate and efficacy of descending glutamatergic inputs from the GRN in the resting and walking mouse. (A, B) Examples of averaged rectified EMG response of the iTA at (A) rest and (B) during locomotion, either during the relaxed phase (“Rel”; stance) or the activated phase (“Act”; swing) upon a 10-ms photostimulation. (C, D) Boxplots of (C) successes and (D) amplitude area (efficacy) of the EMG response for each muscle for the different state of the spinal network. Kruskal-Wallis test and multiple comparisons with Tukey HSD test: *p < 0.05, **p < 0.01, ***p < 0.001. Data can be found in S1 Data. cGL; contralateral gastrocnemius lateralis; cTA, contralateral tibialis anterior; EMG, electromyographic; GRN, gigantocellular reticular nucleus; HSD, honest significant difference; iGL, ipsilateral gastrocnemius lateralis; iTA, ipsilateral tibialis anterior.

**Fig 8:**
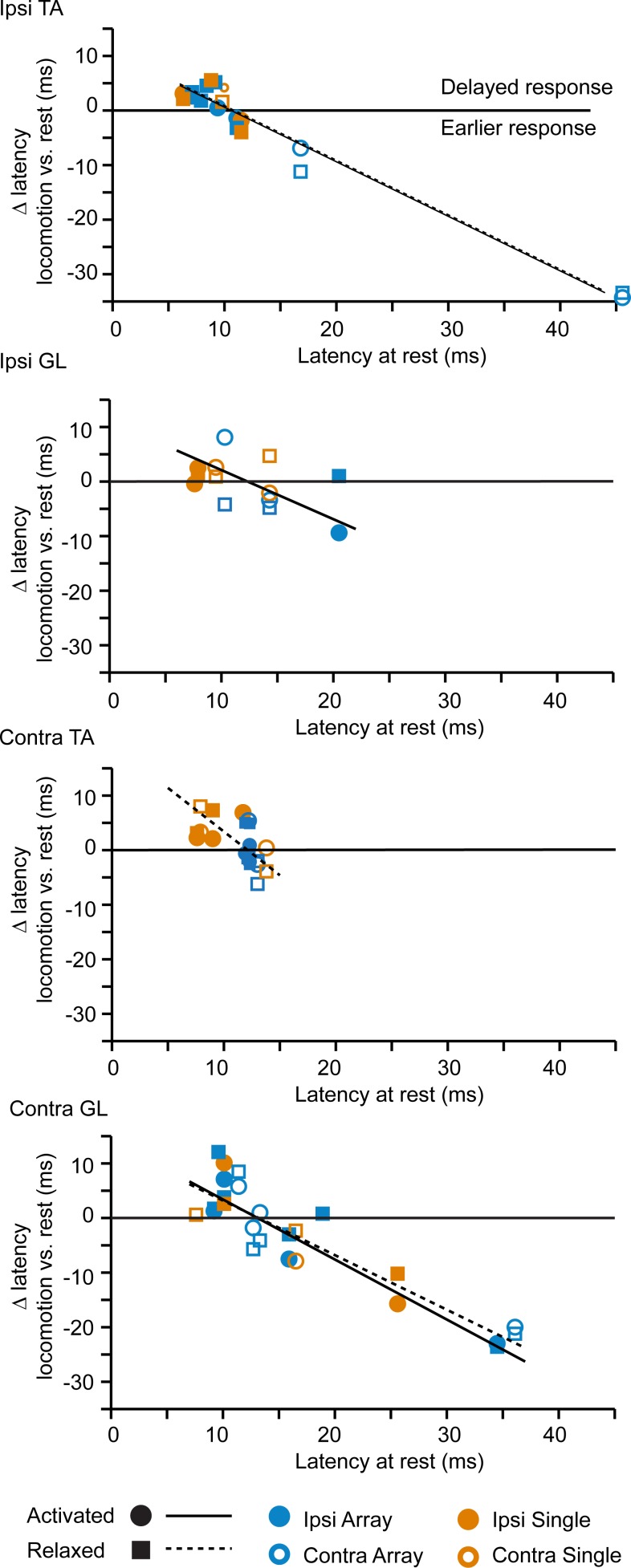
Normalization of the latency of the GRN drive. Linear regression of the variation of latency versus latency at rest: iTA (“Ipsi TA”) during activated state (F-test *p* = 1.6 × 10^−6^, r^2^ = 0.983) and during relaxed state (*p* = 5.8 × 10^−7^, r^2^ = .939); iGL (“Ipsi GL”) during activated state (F-test *p* = 0.033, r^2^ = 0.554) and during relaxed state (*p* = 0.82); cTA (“Contra TA”) during activated state (F-test *p* = 0.334) and during relaxed state (*p* = 0.018, r^2^ = .465); cGL (“Contra GL”) during activated state (F-test *p* = 2.35 × 10^−5^, r^2^ = 0.861) and during relaxed state (*p* = 1.38 × 10^−5^, r^2^ = 0.788). Data can be found in S1 Data. cGL; contralateral gastrocnemius lateralis; cTA, contralateral tibialis anterior; GRN, gigantocellular reticular nucleus; iGL, ipsilateral gastrocnemius lateralis; iTA, ipsilateral tibialis anterior.

Given the absence of long-lasting motor responses during locomotion in comparison to the condition at rest, we hypothesized a gating of descending GRN inputs by the spinal locomotor network. To test this hypothesis, we evaluated the responsiveness (success) and the efficacy (amplitude area of rectified EMG responses) of motor responses evoked in the resting versus walking mouse (Fig 7A and 7B). In comparison to the resting mouse, the ipsilateral flexor (iTA) displayed a smaller probability of motor responses when the muscle was active during locomotion (Fig 7C). Locomotor activity decreased responsiveness in the contralateral flexor regardless of the network state. Regarding efficacy, the amplitude of motor responses of both extensors was significantly reduced during locomotion and especially when these muscles were active (Fig 7D). In addition to the responsiveness and efficacy of motor responses, their latency was also affected by the network state. As shown by linear regression (Fig 8), longer-latency motor responses at rest were shortened during locomotion, whereas shorter-latency motor responses were postponed in all muscles except during the activated states of the cTA and during the relaxed state of the iGL. Taken together, these results suggest a gating of the descending glutamatergic GRN drive during locomotion.

### Long photostimulation fails to initiate locomotion in the resting mouse but resets locomotor rhythm in the walking mouse

**Fig 9:**
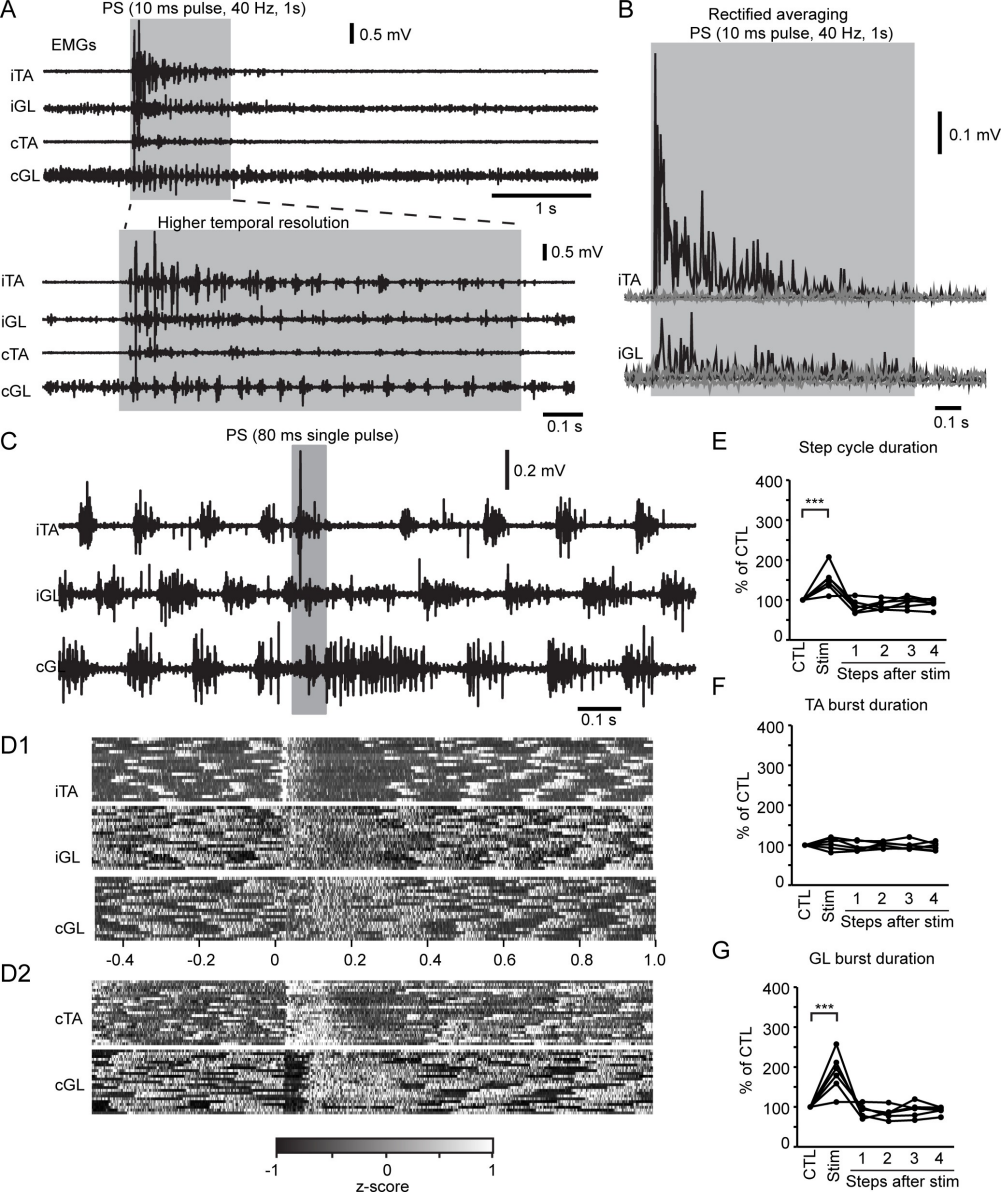
Long PS of VGluT2 GRN neurons reset locomotor rhythm. (A) Low and high temporal resolution of raw EMGs in the mouse at rest. Period highlighted in gray corresponds to long trains of PS (10-ms pulse duration, 40 Hz, 1 s, laser irradiance of 1,130 mW/mm^2^, or 4× threshold). (B) Averaged rectified EMG response of the iTA and iGL illustrated in (A). (C) Raw EMGs during a single pulse of 80-ms PS of VGluT2 GRN neurons during treadmill locomotion. (D) Grayscale-coded matrix of z-transformed EMGs (white, z > 1, black z < −1, gray z ≈ 0). Each row is a PS trial from −0.5 to 1 s after onset of PS. D1, same animal as in (C). D2, another animal illustrating a strong TA activity and inhibited GL activity during PS followed by sustained GL activity and reduced TA activity. (E) Plots of the step cycle duration (expressed as a percentage of CTL, calculated as the 3 steps before stimulation [“stim”]). Each line corresponds to an animal. (F, G) Plots of the (F) TA and (G) GL burst duration. Values are expressed as a % of CTL as in (B). Kruskal-Wallis and post hoc Tukey’s HSD test: ***p < 0.001. Data can be found in S1 Data. cGL, contralateral GL; cTA, contralateral TA; CTL, control; EMG, electromyography; GL, gastrocnemius lateralis; GRN, gigantocellular reticular nucleus; HSD, honest significant difference; iGL, ipsilateral GL; iTA, ipsilateral TA; PS, photostimulation; TA, tibialis anterior; VGluT2, vesicular glutamate transporter 2.

Given the role of descending VGluT2 GRN neurons in locomotor pattern and its flexor-related facilitation, we next hypothesized that these neurons might initiate locomotion in the animal at rest and reset locomotor rhythm during ongoing locomotion. Long pulses or trains of photostimulation (4× threshold or 1,130 mW/mm^2^) gave rise to a high-frequency rhythmic activity, thus recapitulating the motor pattern evoked by short-pulse photostimulation at rest. However, long photostimulation failed to trigger any episodes of locomotion, and the rhythmic activity instead resembled a tremor (Fig 9A and 9B). Varying the intensity (1.5 and 2× threshold) and the frequency (20 and 40 Hz) of photostimulation triggered a similar high-frequency rhythmic activity in recorded hindlimb muscles (S4 Fig) associated with movements of the forelimbs, the trunk, the neck, and the tail.

In contrast, long-pulse photostimulation reset locomotor rhythm during ongoing locomotion (Fig 9C–9G) by stopping and resuming locomotion after a delay [21]. Since the stride duration ranges from 200 to 330 ms at a treadmill speed from 15 to 25 cm/s [22], we set the duration of long photostimulation to 80 ms to alter about 25%–40% of the ongoing step cycle duration without affecting the subsequent step cycles. As shown in Fig 9, 80-ms photostimulation triggered a flexor burst initiating a prolonged step cycle (Fig 9C) due to an increase in the duration of the ongoing extensor burst (Fig 9A, 9B and 9F) in both hindlimbs (Fig 9C and 9D), before resuming a new step cycle at a similar frequency (Fig 9E, similar step cycle duration) to that observed prior to stimulation. The onset of long photostimulation reliably activated flexors, but the burst duration did not outlast the duration of the stimulation, revealing that the longer step cycle was not related to a longer TA burst but a longer GL burst or stance phase (Fig 9C, 9D, 9F and 9G). The normalized amplitude of flexor and extensor bursts was not significantly impaired upon long photostimulation (Kruskal-Wallis, *p* = 0.884 for flexor and *p* = 0.521 for extensor), thus confirming the absence of any spatial recruitment of additional VGluT2 GRN neurons (i.e., no spatial facilitation). Therefore, by prolonging the duration of extensor activity, long activation of VGluT2 GRN reset locomotor rhythm during ongoing locomotion.

### Long photoinhibition of glutamatergic GRN neurons disrupts locomotion

**Fig 10:**
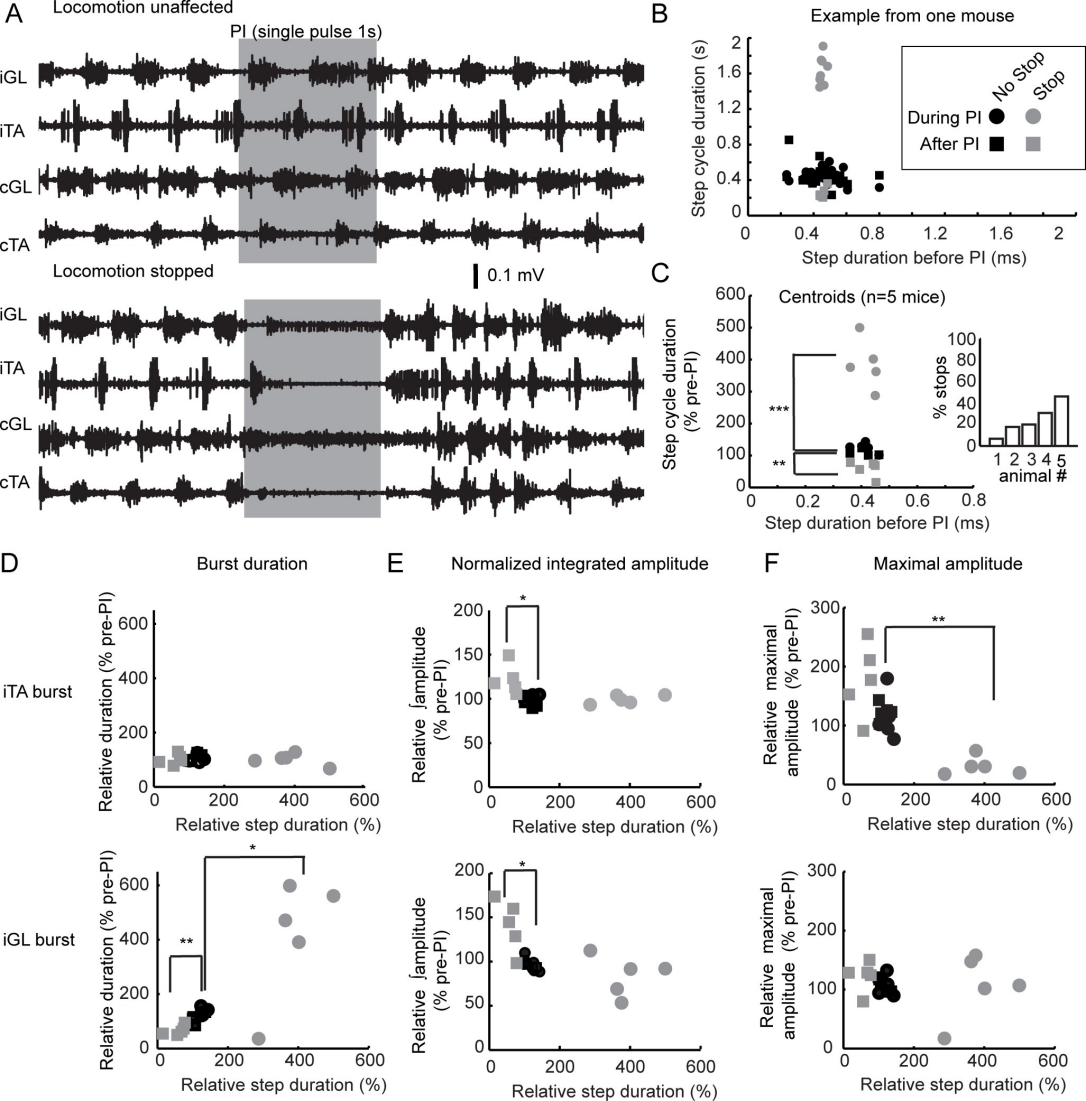
Long PI of VGluT2 GRN neurons in the walking mouse. (A) EMGs of the TAs and GLs during locomotion upon 1-s PI. (B) Plot of the step cycle duration during (circle) and after (square) PI versus the step duration cycle before PI. K-clustering was used to split data based on the step cycle duration during PI. Cycles not affected by PI are in black, and those affected are in gray. Data are from one animal. (C) Left, plot of the step cycle duration during and after PI versus before PI. Each circle/square symbol is a centroid (mean of one mouse). Paired *t* test: ***p < 0.001, **p < 0.01. Right, bar graph of the percentage of step cycles when locomotion was stopped by the PI. (D-F) Plots of centroids of the (D) burst duration, (E) normalized amplitude of the burst, and (F) maximal amplitude of the TA (top) and GL (bottom). Paired *t* test: **p < 0.01, *p < 0.05. Data can be found in S1 Data. cGL, contralateral GL; cTA, contralateral TA; EMG, electromyography; GL, gastrocnemius lateralis; GRN, gigantocellular reticular nucleus; iGL, ipsilateral GL; iTA, ipsilateral TA; PI, photoinhibition; TA, tibialis anterior; VGluT2, vesicular glutamate transporter 2.

Our photostimulation studies demonstrated that VGluT2 GRN neurons are sufficient to modify motor activity as well as locomotor pattern and rhythm. We next tested the hypothesis that they are also necessary. To test this hypothesis, we backcrossed eNpHR3.0-EYFP mice with VGluT2-cre mice (VGluT2-cre::Ai39), allowing us to photoinhibit glutamatergic GRN neurons upon exposure to a yellow laser illumination (594 nm). Short and long photoinhibition pulses were delivered through an optical probe in the GRN at the same level as in our photostimulation experiments. Because short 10-ms pulses of photoinhibition did not affect motor pattern during locomotion (S5 Fig), we used longer pulses. Whereas long photoinhibition (1-s pulse duration) had no effects on muscle tone in the resting mouse (S5 Fig), it impaired locomotor pattern and rhythm in the walking mouse (Fig 10A). The effects were not systematic and were variable from mouse to mouse, either stopping locomotion or not. We used a K-clustering of the step cycle duration during photoinhibition (normalized by the pre-photoinhibition duration) to identify whether locomotion was stopped or not (Fig 10B and 10C, stop and no-stop clusters). Photoinhibition affected 6%–42% of step cycles (Fig 10C). Clustering analysis of data evoked upon 10-ms pulse photoinhibition suggested that 6% of trials affected step cycle duration. However, meaningful clustering was not obtained for 80- and 200-ms pulses of photoinhibition (S5 Fig), suggesting that longer photoinhibition was required to impact locomotion.

In comparison to unaffected bouts of locomotion, transient interruptions (corresponding to a 3- to 5-fold increase in step cycle duration) were followed by episodes of faster stepping after long photoinhibition (Fig 10C). The duration of the extensor burst GL was increased during long photoinhibition and moved below the control baseline thereafter (Fig 10D). Interrupting locomotion for 1 s while the treadmill belt was still running brought the animal to the rear of the treadmill, therefore forcing the animal to accelerate for a few steps to reach the front part of the treadmill belt after photoinhibition. The normalized amplitude of both flexor and extensor muscles was increased after photoinhibition (Fig 10E), and the maximal amplitude of the flexor but not the extensor was reduced during photoinhibition (Fig 10F). Overall, although the effect was variable, long photoinhibition modified locomotor pattern and rhythm during ongoing locomotion, in some cases temporarily halting steady-state locomotion on a treadmill.

## Discussion

Combining EMG recordings with unilateral optogenetic manipulations enabled us to dissect the functional contribution of glutamatergic neurons of the GRN to motor and locomotor activity in the resting and walking mouse. Our results reveal that glutamatergic GRN neurons modulate locomotor pattern and rhythm in the freely behaving mouse, and their descending drive is gated by the locomotor network state. Although unilateral activation with an optical probe of 100 μm of glutamatergic GRN neurons failed to initiate locomotion, it reset locomotor rhythm. Inhibition could stop it, albeit with variability.

### Methodological considerations

In this study, we investigated the impact of the descending drive from glutamatergic GRN neurons on the lumbar spinal network. We did not attempt to differentiate the contribution of direct reticulospinal projections to lumbar interneurons from that of indirect reticulospinal projections (i.e., via descending propriospinal neurons or the cervical network) or from the reticular formation network itself. As such, we have avoided using the term “reticulospinal” to describe the targeted population of the GRN.

EMG recordings enabled us to monitor, at a high spatiotemporal resolution, motor changes that could not have been addressed with a sole kinematic analysis. Photostimulation was set at the minimum laser irradiance necessary to evoke motor transients, thus preventing any gross changes in behavior or locomotor gait. Photostimulation (or photoinhibition) is likely restricted to the area lying below the tip of the optical probe. Calculations derived from brain measurements [23] suggest that irradiance at 1-mm depth has already decreased by 99%. Although we cannot exclude a potential recruitment of the dorsal part of the ventral/alpha portion of the GRN, the primary population recruited still remained the GRN. Because of the possibility of antidromic photoactivation, upstream neural circuits presynaptic to the GRN may have contributed to a confounding superimposition of motor effects through other spinal, brainstem, midbrain, or cortical regions. However, previous studies using photostimulation varying from milliseconds to a second evoked only single antidromic action potentials, with low reliability. Moreover, long photostimulation up to 1 s never triggered any tonic activation of presynaptic populations rostral to the photostimulated region [24, 25]. Taken together, these findings argue that motor and locomotor responses evoked upon our optogenetic manipulations reflected mainly changes in the synaptic efficacy of glutamatergic GRN neurons.

### Gating of the descending glutamatergic GRN drive

Despite long-lasting motor responses at rest, we found a lower responsiveness and efficacy to short-pulse photostimulation during locomotion. The low amplitude and probability of motor responses to short-pulse photostimulation during locomotion might reflect a gating of glutamatergic GRN neurons. Microstimulation studies using 30-ms trains have found robust responses in the same short-latency range as those evoked by our short-pulse photostimulation of 10 ms [11, 13], therefore suggesting reduced temporal facilitation in our study. With this reduced facilitation, the lower responsiveness of the spinal network to the glutamatergic brainstem drive might hence reflect the probabilistic nature of transmitter release [26, 27], which might fluctuate with the level of spontaneous network activity [28]. As previously reported in the brain [29, 30], an increased conductance in spinal interneurons decreases input resistance by 35% during locomotor-like activity in neonatal rats [31]. A lower input resistance during network activity underlying motor activity is accompanied by a decrease in excitability of spinal interneurons and motoneurons [32]. Such mechanisms could contribute to a gating of the descending glutamatergic drive.

### Motor and postural tone in the resting and walking animal

As shown in previous microstimulation studies in the resting animal [33, 34], our photostimulation evoked motor increases in both flexors and extensors, with the shortest latency and the strongest amplitude in the ipsilateral flexor. These motor effects recapitulate, to some extent, the strong postsynaptic calcium transients evoked in flexor-related motoneurons upon electrical microstimulation of the brainstem in neonatal mouse preparations [35, 36]. In contrast to decerebrated animal preparations characterized by motor rigidity [37–40], but in agreement with previous electrical microstimulation studies in freely behaving animals [11, 13], both short (10-ms) and long (80-ms) photostimulation usually facilitated motor responses by increasing the motor drive. Nevertheless, long photoinhibition (1 s) failed to impair muscle tone, thus suggesting that glutamatergic GRN neurons likely play an important, but not essential, role in maintaining motor tone.

### Flexor- versus extensor-related locomotor activity

Like previous microstimulation studies in decerebrated, thalamic, and intact animals [11–14], short-pulse photostimulation of glutamatergic GRN neurons also evoked a reciprocal coactivation in antagonist muscles during the ipsilateral swing phase, thus likely securing the hindlimbs during the swing phase, whereas it clearly exhibited a flexor-predominant facilitation during the stance phase with a strong motor increase in flexors and an inhibition in extensors, thus likely contributing to the coming swing phase of the next step cycle. A similar phase-dependent locomotor pattern was also evoked in contralateral hindlimb muscles with a significantly longer latency, likely resulting from a polysynaptic pathway through spinal commissural interneurons [41–44]. Although most reticular and reticulospinal neurons discharge more often in phase with extensors than flexors, about a quarter of reticulospinal neurons have been shown to discharge with a double burst in phase with ipsilateral and contralateral flexors during locomotion [7], thus suggesting that these double-burst GRN neurons might be glutamatergic.

Similarly, long photostimulation of 80-ms pulse duration evoked an early flexor facilitation followed by a sustained extensor facilitation, thus increasing extensor burst duration and delaying the onset of the next flexor burst during ongoing locomotion. As recently reported for optogenetic activation of a subpopulation of glutamatergic GRN neurons using neonatal mouse preparations [16], such a temporal facilitation of the extensor pool could disfacilitate flexor motoneurons. Alternatively, given that most reticular and reticulospinal neurons discharge in phase with extensors [7], long photostimulation could also temporally recruit new populations, enhancing a tonic drive onto extensors in response to unexpected perturbations during locomotion [11, 13].

Conversely, long photoinhibition disrupted locomotor pattern and rhythm by reducing the recruitment of flexor and extensor motor pools and by prolonging the extensor phase, thus eventually halting locomotion. This locomotor arrest was unlikely, because of a reversal potential of chloride-conducting halorhodopsins [45]. Therefore, our optogenetic studies argue that glutamatergic GRN neurons drive a short-latency flexor-related activity and a long-latency extensor activity.

### Resetting: Initiating or halting locomotion?

A resetting of locomotor rhythm has been previously reported upon long trains of microstimulation of the GRN during fictive and decerebrated locomotion [12, 14, 46, 47]. Since a locomotor reset starts with an interruption of locomotor rhythm [21], our results argue that glutamatergic GRN neurons have access to the rhythm generator and might be considered “stop cells.”

Similar to findings from in vitro studies in neonatal rodents [48, 49] or lower vertebrates [50, 51], our long photostimulation of glutamatergic GRN neurons evoked a high-frequency rhythm in the resting mouse more closely resembling a reciprocal co-contraction of flexor–extensor muscles than locomotion. As previously reported [10, 13, 52, 53], these transient reciprocal coactivations might have functional relevance during postural adjustments while reaching an object, stepping over an obstacle, or losing ground support.

On the other hand, some brainstem neurons have been reported to control the termination of locomotion or a pause prior to an escape behavior in lower vertebrates [54–56]. Similar to the findings of a recent study targeting V2a GRN neurons [16], our photostimulation of glutamatergic GRN neurons prolonged the extension phase. Because most of our experiments were performed at steady speed during treadmill locomotion, our results argue for a “pause” rather than a “stop” command for postural adjustments. This argument is supported by the fact that photoinhibition of glutamatergic GRN disrupted locomotor pattern and rhythm, thus halting locomotion. In addition, our EMG analysis reveals in detail that these changes were not due to either muscle tone inhibition, as previously reported in decerebrated animal studies [57, 58], or a reciprocal coactivation of antagonist flexor–extensor muscles, as expected during a freezing of gait [59, 60].

### Functional contribution of glutamatergic versus V2a GRN neurons

Although a majority of V2a GRN neurons are glutamatergic, not all glutamatergic GRN neurons might be V2a, and not all V2a project in the spinal cord: some project locally within the brainstem and presumably within the GRN [15, 16, 18, 61].

Previously, we have shown that V2a GRN neurons are glutamatergic; some of them are reticulospinal and express c-FOS (a cell activity marker) following an episode of locomotion, thus suggesting that they are active during walking [15]. Furthermore, these V2a neurons also receive anatomical and functional inputs from locomotor-related midbrain nuclei and have electrophysiological properties supporting tonic repetitive firing. Recently, it has been shown that long trains of photostimulation of 20–40 Hz for 500 ms to 1 s can stop ongoing locomotion in a corridor, thus suggesting that V2a GRN neurons are stop cells [16]. Using a different paradigm (treadmill locomotion versus overground locomotion), shorter stimulation parameters (single pulses of 80 ms versus long trains up to 1 s), and activation of a smaller region (unilateral versus bilateral stimulation of the GRN), we also found that photoactivation of VGluT2 GRN neurons could reset and therefore to some extent stop locomotion, thus arguing that VGluT2 GRN neurons, like V2a GRN neurons, could be considered stop cells.

More recently, long trains of photostimulation of 100 Hz for 5 s delivered above VGluT2 GRN neurons have been shown to induce head turnings alone, with no effects on locomotor speed [19]. Similarly, our long pulses of photostimulation did not modify overall locomotor frequency. However, they reset the locomotor rhythm of the stimulated step cycle, thus showing that VGluT2 GRN neurons have access to the rhythm generator. Regarding head turnings, our electrophysiological recordings show that GRN neurons fired action potentials in the 20-Hz range and collapsed at 40 Hz and beyond, therefore suggesting that high-frequency stimulations of 100 Hz might alter the motor output of the GRN, contributing to head turnings. Nevertheless, as previously reported by electrical microstimulation studies [34], long trains of photostimulation of VGluT2 GRN neurons also evoke complex motor and postural movements from the neck, tail, forelimb, and hindlimb, according to previous videos [19], and our EMG recordings also showed hindlimb muscles activation at rest and during locomotion.

As previously shown in the crayfish, tadpole, cricket, and lamprey [54, 55, 62, 63], further electrophysiological studies will be necessary to document the firing pattern of V2a and VGluT2 GRN neurons to determine more clearly the functional contribution of these neuronal populations and their reticulospinal counterpart in initiating, maintaining, and stopping locomotion in freely behaving mice.

In conclusion, our results reveal a critical role of glutamatergic GRN neurons in maintaining a descending tonic drive on motor tone in addition to phasically modulating motor and locomotor activity in flexor and extensor muscles during locomotion, presumably through the spinal interneuronal circuit. Although photostimulation of glutamatergic GRN neurons failed to initiate locomotion, they contributed to locomotor pattern and they reset locomotor rhythm (and probably even stopped it), and photoinhibition could halt locomotion. Such a reset likely enables a pause, allowing postural adjustments during unexpected perturbations or goal-directed locomotion.

## Materials and methods

### Ethics statement

All animal experiments were approved by the Animal Welfare Committee of CHU de Québec and Université Laval (approval number 15–007 and 15–020) in accordance with the Canadian Council on Animal Care policy.

### Surgery

Experiments were conducted on VGluT2-ires-cre mice (RRID: IMSR_JAX:016963) of either sex (>2 mo), in which ChR2 was expressed by crossing Ai32 (ChR2[H134R]-EYFP, Jackson Laboratory; RRID:IMSR_JAX:012569) reporter mice, whereas Halorhodopsin (eNpHRv3.0) was expressed by crossing Ai39 (RCL-eNpHR3.0/EYFP, Jackson Laboratory; RRID:IMSR_JAX:014539) reporter mice. Mice were anesthetized with isoflurane (2%–3%), and all pressure points and sites of incision were injected with lidocaine-bupivacaine. Analgesia (buprenorphine) was given at the beginning of the surgery. Animals were placed into a stereotaxic frame and kept warm on a heating blanket. A craniotomy was performed above coordinates of interest (bregma −5.9 to −6.9 mm, mediolateral 0.4 to 0.9 mm, depth from brain surface 4.4 to 5.0 mm). The bregma, mediolateral, and depth were adjusted to target the center of the GRN. Optical probes (single fiber of 100 μm in diameter or array of 3 fibers each spaced by 200 μm) were inserted at coordinates of interest. For photoinhibition experiments, all optical probes were implanted from bregma −6.0 mm, mediolateral 0.4 mm, and depth 5.0 mm. Probes were secured with a metal screw fixed in the skull and dental cement. Analgesia (carprofen) was given for 3 d after surgery. Mice were allowed 1 wk to recover before we performed electrophysiological recordings. Mice were housed separately, fed ad libitum, and followed a normal 12-h light/dark cycle.

### Treadmill locomotion

Mice were trained to walk on a single-lane mouse treadmill (Panlab, Barcelona, Spain) at 15–20 cm/s. Mice that did not maintain position on the treadmill, either by staying at the rear or jumping at the front, were excluded. Four animals out of 20 were excluded. Mice were not restrained except by the optical patchcord and the EMG wires, which were held high and were long enough not to generate any strain. On the first day of training, an electrical grid was used to motivate the animal into walking. Mice learned the task very quickly (2–3 trials) to avoid the rear of the treadmill. The electrical grid was then turned off during trials. Furthermore, the treadmill was stopped when the animal maintained position on the treadmill to positively reinforce the behavior. A few minutes of rest were granted between trials of 3–5 min.

### Histology

At the end of the experiment, mice were perfused with ice-cold PFA 4%. The brain was extracted, frozen, cryosectioned, and mounted on slides for immunostaining. VGluT2 neurons were labeled with anti-Cre recombinase (1:3,000, Abcam; Cat# ab190177). Fluorescent secondary antibodies AlexaFluor-488 or AlexaFluor-594 were used for anti-Cre (1:1,000, Invitrogen). EYFP signal was amplified with anti-GFP antibodies (1:1,000, Abcam; Cat# ab13970), and AlexaFluor-488 was used as a secondary antibody. Sections were visualized using an LSM 800 laser scanning confocal microscope. Images were acquired and processed with Zen software (Zeiss).

The track of the optical probe was found on brain sections, and the deepest point was determined as the tip. We evaluated whether distances from the pial surface match those calculated during surgery. Because the brainstem barely changes over the anteroposterior axis for our targeted region (AP −5.7 and −6.9), anteroposterior coordinates reported in Fig 1D were those calculated during surgery. Anatomical landmarks were used to evaluate the correct position of the tip: dorsally, the shape of the fourth ventricle and the dense cre-recombinase signal of the vestibular nuclei; medially, the medial longitudinal fascicle; ventrally, the pyramidal tract and dense cre-recombinase signal of the GRN pars alpha; laterally, the trigeminal complex. The tip was located in a region with large cell bodies of Cre-positive neurons.

### Electrophysiological experiments in anesthetized mice

Mice were anesthetized with ketamine (100 mg/kg, i.p.) and xylazine (10 mg/Kg, i.p.). The mouse was placed in a stereotaxic frame, and the cerebellum was exposed by a craniotomy and durectomy. A 0.1 MΩ tungsten electrode (FHC, Bowdoin, ME, United States) glued with epoxy to an optical fiber (diameter 100 μm, 0.37 NA) was lowered in the GRN (bregma −6 mm, mediolateral 0.4 mm, depth 5.0 mm). The tip of the electrode was slightly lower (about 0.2–0.3 mm) than the optical fiber. Photostimulation (13 mW) was delivered in single pulses (10 ms and 1 s) and trains (10 ms, 20 and 40 Hz). EMGs from the TA and the GL of the ipsilateral hindlimb were recorded simultaneously. To evaluate the contribution of local glutamatergic transmission in mediating MUA and EMG responses, 150 nL CNQX (100 M, Sigma Cat#C239) and AP-5 (500 M, Sigma Cat#5282) dissolved in PBS 0.1 M were injected locally (about 0.2–0.3 mm from the optrode tip) with a nanoliter injector (Nanoliter2010, WPI, Sarasota, FL, US). The signal was amplified (Extracellular Amplifier Model 1800, A-M Systems, Sequim, WA, US) using a high-pass filter (300 Hz) to isolate multiunitary activity and digitized (Power1401-3, CED, Cambridge, United Kingdom) for offline analysis on Spike2 (CED, Cambridge, UK).

### Electrophysiological experiments in freely behaving mice

As described previously [22], we recorded the EMG activity of the ankle flexor (TA) and ankle extensor (GL) with single-strand nickel-chromium wire (50-μm diameter) arranged in a duplex configuration (California Fine Wire, catalog# CFW2027239). For acute EMG insertion, the animal was anesthetized with isoflurane (1%–2%). The hindlimb was shaven and the skin cleaned with alcohol to clearly identify muscles. EMG wires were inserted percutaneously with a 27G needle aligned parallel to muscles. A copper ground was inserted below the skin of the lower back. All wires were attached together with adhesive tape. At the end of the recording session, wires were removed under isoflurane anesthesia. No analgesia was provided during or after EMG sessions. Mice were observed 1 h after the recording session for any signs of pain or distress (which were never observed). The duplex configuration allows EMG recordings with low background noise. Differential amplification provides recordings free of movement artefacts. Background EMG activity and EMG responses evoked upon photostimulation (Blue DPSS laser, LRS-0473-PFM, Laserglow Technologies) or photoinhibition (Yellow DPSS laser, LRS-09589, Laserglow Technologies) were recorded at rest and during treadmill locomotion at 15–30 cm/s. We always used 10-ms pulse duration except when we investigated the role of glutamatergic neurons of the GRN in initiating locomotion (1-s trains) and resetting locomotor rhythm (80-ms pulse), as well as the impact of photoinhibition (1-s pulse). The EMG signal was amplified (×1,000) with custom-built extracellular amplifiers, band-pass filtered (0.1–10,000 Hz), digitally converted (Power 1401, CED, UK), and stored with Spike 2 version 8 (CED, UK; RRID:SCR_000903) for offline analysis.

### Data analysis

As previously described [64], EMG data were analyzed with custom-designed software and Matlab (MathWorks). To evaluate EMG activity and response during locomotion, only bouts of stable locomotion (at least 8 continuous regular steps, including 3–4 before the stimulated step cycle) when the animal was in the front third of the treadmill lane were analyzed. To evaluate the averaged locomotor burst, the number of unstimulated steps was twice the number of stimulated steps. Only muscles with clearly identifiable bursts were analyzed. Latency, duration, and integrated amplitude were extracted from rectified EMG. The distributions were first evaluated with histograms (number of bins determined by Sturges’ rule). When we observed bi- or trimodal distribution, we used K-means clustering to split data. In the case of bimodal distribution, values clustered around zero were considered as failure to induce a detectable response. In the case of trimodal distribution, the highest values were considered outliers if they lay outside the Tukey boxplot (upper quartile + 1.5 time the interquartile range). Outliers were considered successes but were excluded from the averaging. Success probability was calculated as the number of successes out of the total number of stimulations and expressed as a percentage. Latency was defined as the time when the averaged EMG response crossed the positive 99.95% interval of confidence of baseline (nonstimulated bouts of recording) amplitude. Total duration corresponded to the interval of time between latency and recrossing. In some cases, a pause of 40 ms or less was observed. Because a second motor response of substantial duration followed this short pause, total duration was evaluated as the second crossing below the 99.95% interval of confidence (Fig 3C). Integrated amplitude for the equivalent duration was measured as the area under the curve. Relative amplitude for each animal was obtained by dividing the integrated amplitude of each period by the maximal absolute value.

### Statistics

To compare success, latency, amplitude, and duration of EMG responses at different intensities of photostimulation, we used either an ANOVA (parametric) or a Kruskal-Wallis test (nonparametric). The choice of a parametric versus nonparametric test depended on the heteroscedasticity of variances as evaluated by a Bartlett’s test. Post hoc paired comparisons were performed with a *t* test with Bonferroni correction after an ANOVA or a Tukey’s honest significant difference (HSD) test after a Kruskal-Wallis. The relation between the variation of latency during locomotion versus rest was evaluated with a linear regression.

## Supporting information

S1 Data (XLSX)

S2 Data (XLSX)

S1 Fig Types of probes used and calculation of irradiance. Schema of types of probes (single fiber versus array of triple fibers) and calculations for the irradiance of these probes.
(TIF)

S2 Fig Orthogonal projections of ChR2–EYFP and Cre-recombinase signal in VGluT2 GRN neurons. Confocal images with orthogonal views of Cre-positive neurons (red) and ChR2–EYFP fusion protein (green) in the GRN. ChR2–EYFP is observed on the membrane of the Cre-positive neurons but also on presumed dendrites and axons. ChR2, channelrhodopsin-2; EYFP, enhanced yellow fluorescent protein; GRN, gigantocellular reticular nucleus; VGluT2, vesicular glutamate transporter 2.
(TIF)

S3 Fig The effect of photostimulating VGluT2 neurons of the GRN are not affected by blocking local glutamatergic transmission with CNQX and AP-5. (A) Raster plot of 200 consecutive photostimulations (10-ms pulse, every 3 s for 10 min). First pulse starts right after the end of CNQX 100 μM and AP-5 500 μM injection (150 nL infused at a rate of 2 nL/s) with a glass pipette inserted approximately 0.3 mm away from the optrode (100-μm optical fiber 0.37 NA glued to a tungsten electrode 0.1 MΩ). Despite a very small change in timing, pharmacological blockade did not prevent firing upon photostimulation. (B) Traces of MUA from the GRN and EMG from the TA and GL during the first and last pulse. Response was not abolished by pharmacological blockade. (C) Rectified EMGs during the first and last 10-ms pulses were used to calculate in (D) the integrated amplitude of the EMG response evoked by photostimulations. (D) Plots of the spikes per second in the GRN (top), the maximal amplitude (middle), and integrated amplitude (bottom) of the rectified EMG versus time from the end of injection. Pharmacological blockade did not abolish the firing and EMG response over time. (E) Mean maximal amplitude and integrated amplitude (area) of the TA and GL during the first and last minutes of the recording illustrating the absence of change following pharmacological blockade. These data argue for a direct effect of photostimulation on motor response. Data can be found in S2 Data. AP-5, (2R)-amino-5-phosphonovaleric acid; CNQX, 6-cyano-7-nitroquinoxaline-2,3-dione; EMG, electromyography; GL, gastrocnemius lateralis; GRN, gigantocellular reticular nucleus; MUA, multiunitary activity; TA, tibialis anterior; VGluT2, vesicular glutamate transporter 2.
(TIF)

S4 Fig PS of 1 s at rest. EMG of the TA and the GL ipsilateral (iTA, iGL) and contralateral (cTA, cGL) to the optical implant in the GRN. Pulses of 10 ms were delivered for 1 s at 20 Hz (left) and 40 Hz (right). Laser intensity was 1.5× threshold (top) or 2× threshold (bottom). Motor response was evoked and consisted of a co-contraction of hindlimb muscles (EMG) accompanied by forelimb, trunk, tail, and neck movements (data not shown). EMG, electromyography; GL, gastrocnemius lateralis; PS, photostimulation; TA, tibialis anterior.
(TIF)

S5 Fig PI at rest and shorter-duration pulses during locomotion. (A) EMG of the GL and TA at rest. A single pulse (1 s) of PI has no effect, and no rebound is observed upon cessation of the light pulse. (B) Averaged rectified EMG of the GL and the TA for each of the 5 periods of the step cycle (triggered on the onset of the stance phase). Gray boxes highlight the 10-ms single pulse of PI. No effect was observed. (C) Clustering analysis applied to a single pulse of PI of 10, 80, and 200 ms in duration. A pulse of 10 ms prolonged the ongoing step cycle in 2 out of 33 cases (6.1%). Clusters during 80 and 200 ms were due to step cycle duration variability being larger before PI than during PI. For this reason, we opted to use longer pulses (1 s) of PI during locomotion to obtain a more robust effect. Data can be found in S2 Data. EMG, electromyography; GL, gastrocnemius lateralis; PI, photoinhibition; TA, tibialis anterior.
(TIF)

## Abbreviations

AP-5: (2R)-amino-5-phosphonovaleric acid
a.u.: arbitrary units
cGL: contralateral GL
ChR2: channelrhodopsin-2
CN: cochlear nucleus
CNQX: 6-cyano-7-nitroquinoxaline-2,3-dione
cTA: contralateral TA
EGFP: enhanced green fluorescent protein
EMG: electromyographic
EYFP: enhanced yellow fluorescent protein
GFP: green fluorescent protein
GL: gastrocnemius lateralis
GRN: gigantocellular reticular nucleus
IC: inferior colliculus
icp: inferior cerebellar peduncle
iGL: ipsilateral GL
iTA: ipsilateral TA
ML: medial lemniscus
TA: tibialis anterior
VGluT2: vesicular glutamate transporter 2
z-diff: z-difference
