# Supplementary figures and images for: Glutamatergic neurons of the gigantocellular reticular nucleus shape locomotor pattern and rhythm in the freely behaving mouse
Source: PLoS Biol. 2019 Apr 24;17(4):e2003880. doi: 10.1371/journal.pbio.2003880 (PMC6502437; doi:10.1371/journal.pbio.2003880)

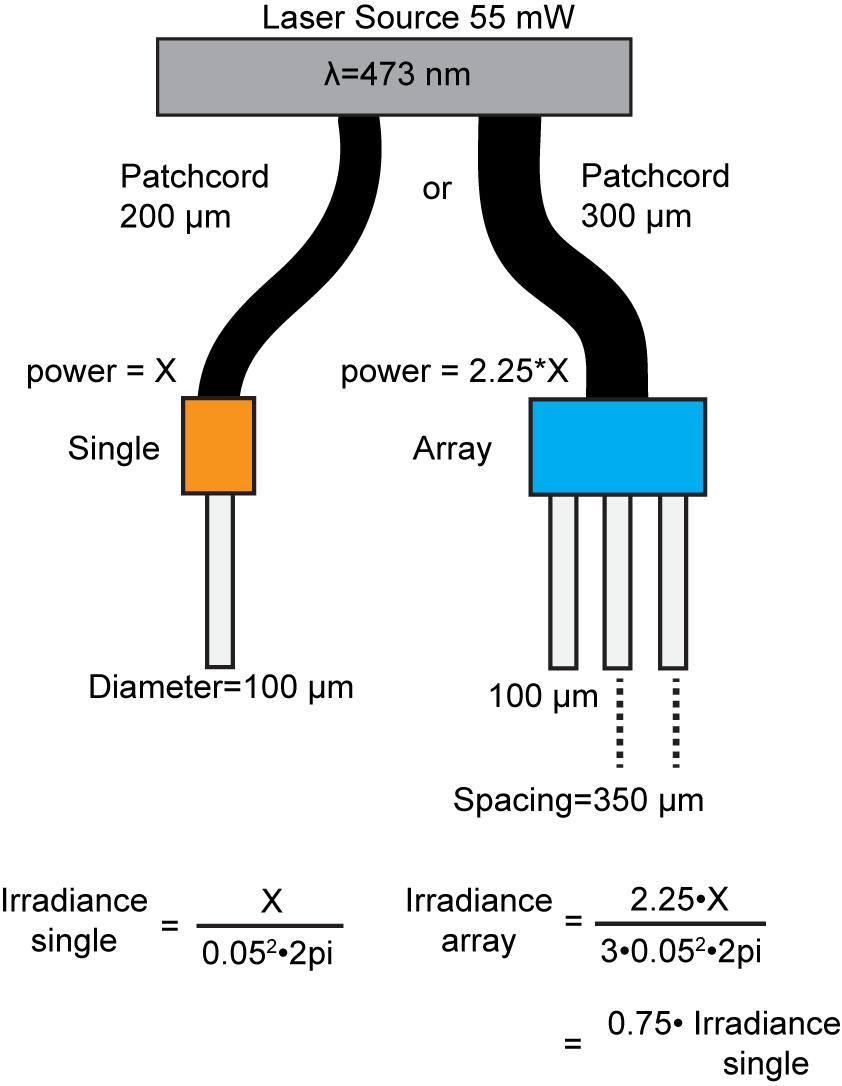

Supplement: S1 Fig — Schema of types of probes (single fiber versus array of triple fibers) and calculations for the irradiance of these probes. (TIF) [file pbio.2003880.s003.tif]

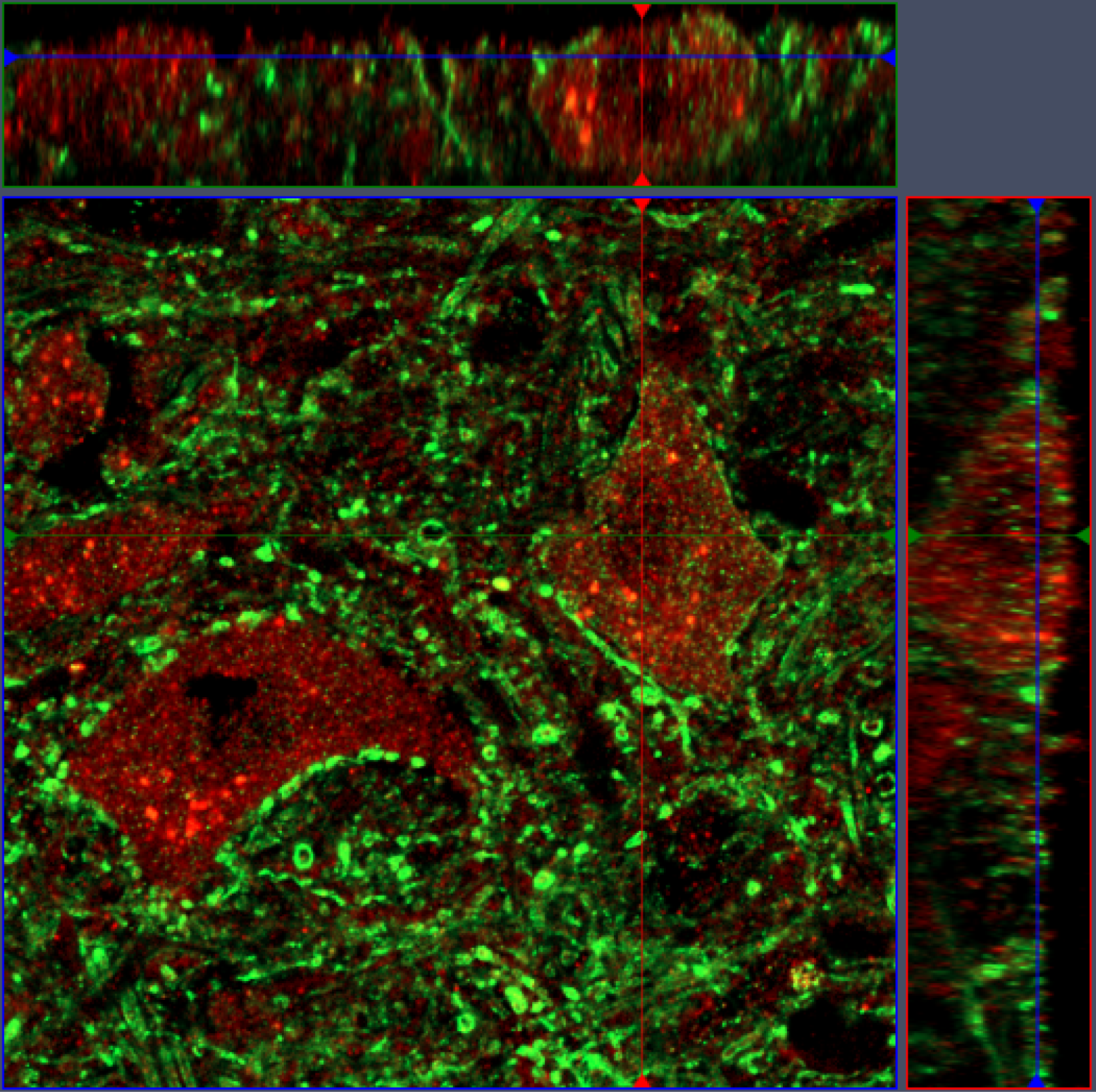

Supplement: S2 Fig — Confocal images with orthogonal views of Cre-positive neurons (red) and ChR2–EYFP fusion protein (green) in the GRN. ChR2–EYFP is observed on the membrane of the Cre-positive neurons but also on presumed dendrites and axons. ChR2, channelrhodopsin-2; EYFP, enhanced yellow fluorescent protein; GRN, gigantocellular reticular nucleus; VGluT2, vesicular glutamate transporter 2. (TIF) [file pbio.2003880.s004.tif]

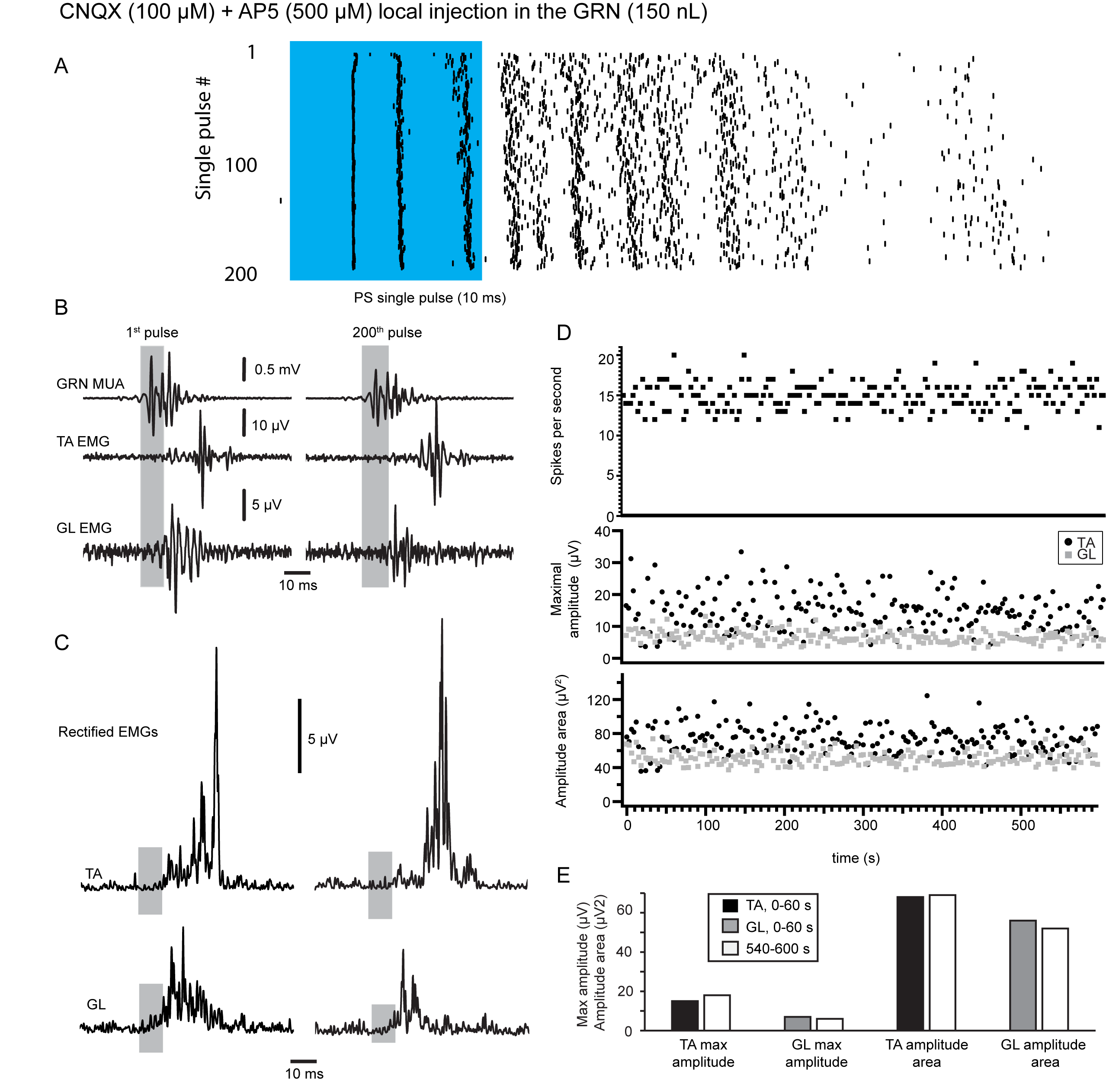

Supplement: S3 Fig — (A) Raster plot of 200 consecutive photostimulations (10-ms pulse, every 3 s for 10 min). First pulse starts right after the end of CNQX 100 μM and AP-5 500 μM injection (150 nL infused at a rate of 2 nL/s) with a glass pipette inserted approximately 0.3 mm away from the optrode (100-μm optical fiber 0.37 NA glued to a tungsten electrode 0.1 MΩ). Despite a very small change in timing, pharmacological blockade did not prevent firing upon photostimulation. (B) Traces of MUA from the GRN and EMG from the TA and GL during the first and last pulse. Response was not abolished by pharmacological blockade. (C) Rectified EMGs during the first and last 10-ms pulses were used to calculate in (D) the integrated amplitude of the EMG response evoked by photostimulations. (D) Plots of the spikes per second in the GRN (top), the maximal amplitude (middle), and integrated amplitude (bottom) of the rectified EMG versus time from the end of injection. Pharmacological blockade did not abolish the firing and EMG response over time. (E) Mean maximal amplitude and integrated amplitude (area) of the TA and GL during the first and last minutes of the recording illustrating the absence of change following pharmacological blockade. These data argue for a direct effect of photostimulation on motor response. Data can be found in S2 Data. AP-5, (2R)-amino-5-phosphonovaleric acid; CNQX, 6-cyano-7-nitroquinoxaline-2,3-dione; EMG, electromyography; GL, gastrocnemius lateralis; GRN, gigantocellular reticular nucleus; MUA, multiunitary activity; TA, tibialis anterior; VGluT2, vesicular glutamate transporter 2. (TIF) [file pbio.2003880.s005.tif]

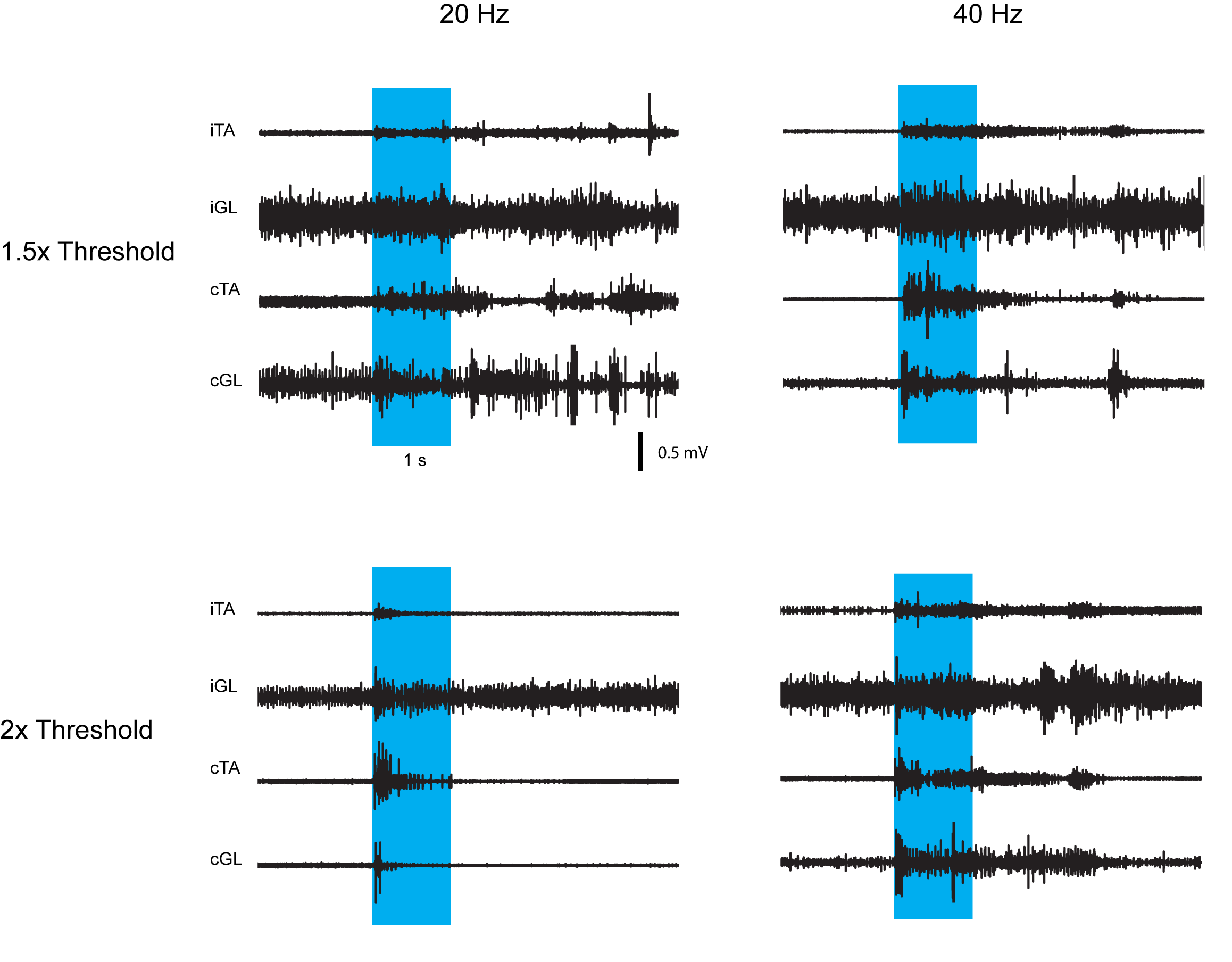

Supplement: S4 Fig — EMG of the TA and the GL ipsilateral (iTA, iGL) and contralateral (cTA, cGL) to the optical implant in the GRN. Pulses of 10 ms were delivered for 1 s at 20 Hz (left) and 40 Hz (right). Laser intensity was 1.5× threshold (top) or 2× threshold (bottom). Motor response was evoked and consisted of a co-contraction of hindlimb muscles (EMG) accompanied by forelimb, trunk, tail, and neck movements (data not shown). EMG, electromyography; GL, gastrocnemius lateralis; PS, photostimulation; TA, tibialis anterior. (TIF) [file pbio.2003880.s006.tif]

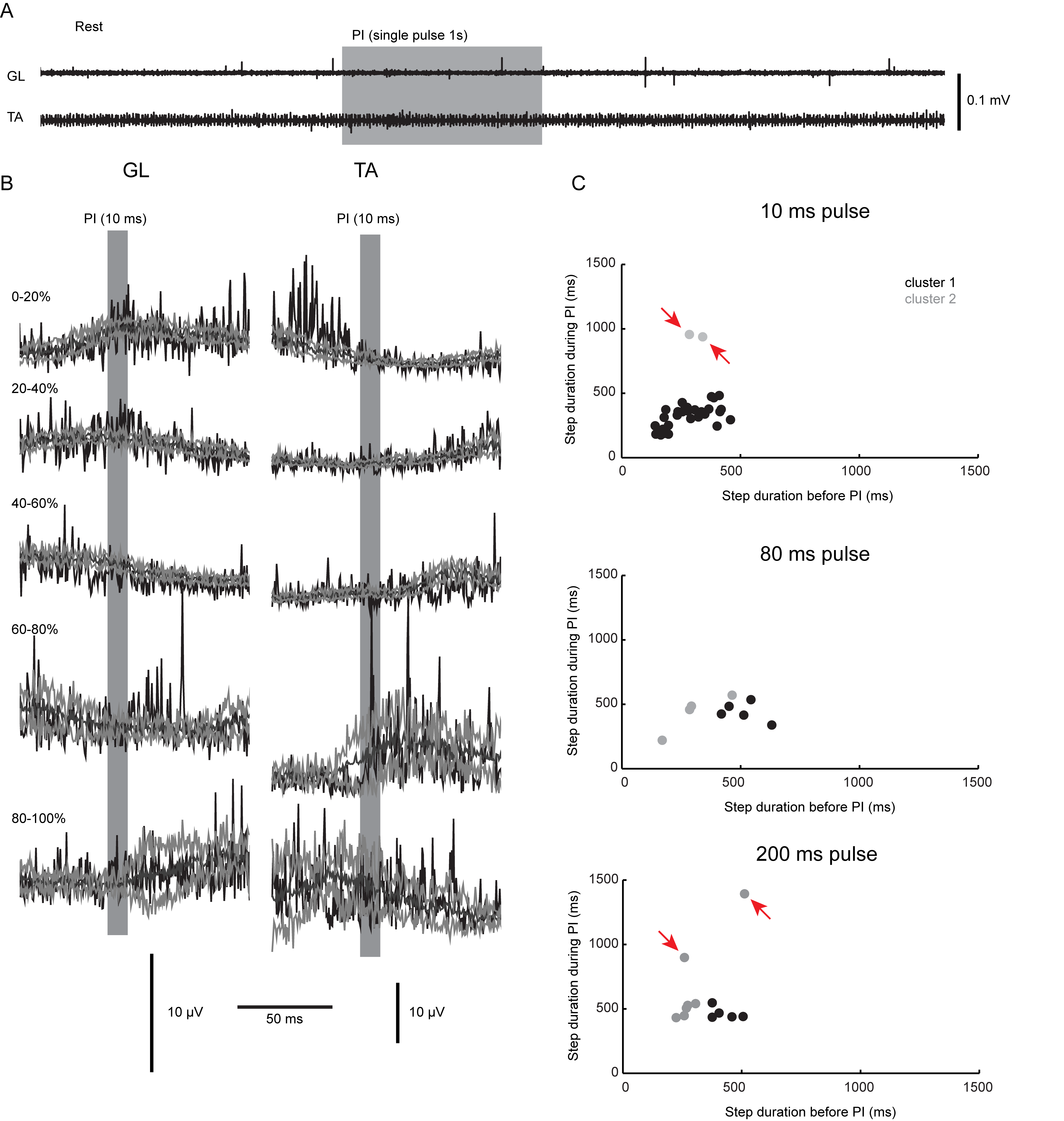

Supplement: S5 Fig — (A) EMG of the GL and TA at rest. A single pulse (1 s) of PI has no effect, and no rebound is observed upon cessation of the light pulse. (B) Averaged rectified EMG of the GL and the TA for each of the 5 periods of the step cycle (triggered on the onset of the stance phase). Gray boxes highlight the 10-ms single pulse of PI. No effect was observed. (C) Clustering analysis applied to a single pulse of PI of 10, 80, and 200 ms in duration. A pulse of 10 ms prolonged the ongoing step cycle in 2 out of 33 cases (6.1%). Clusters during 80 and 200 ms were due to step cycle duration variability being larger before PI than during PI. For this reason, we opted to use longer pulses (1 s) of PI during locomotion to obtain a more robust effect. Data can be found in S2 Data. EMG, electromyography; GL, gastrocnemius lateralis; PI, photoinhibition; TA, tibialis anterior. (TIF) [file pbio.2003880.s007.tif]
